# Activating the d-Tagatose Production Capacity of *Escherichia coli* with Structural Insights into C4 Epimerase Specificity

**DOI:** 10.1021/acs.jafc.4c12842

**Published:** 2025-02-25

**Authors:** Dileep Sai Kumar Palur, Jayce E. Taylor, Bryant Luu, Ian C. Anderson, Augustine Arredondo, Trevor Gannalo, Bryan A. Skorka, Pamela R. Denish, John Didzbalis, Justin B. Siegel, Shota Atsumi

**Affiliations:** † Department of Chemistry, University of California, Davis, Davis, California 95616, United States; ‡ Biochemistry, Molecular, Cellular, and Developmental Graduate Group, University of California, Davis, Davis, California 95616, United States; § Integrative Genetics and Genomics, University of California, Davis, Davis, California 95616, United States; ∥ Biophysics Graduate Group, University of California, Davis, Davis, California 95616, United States; ⊥ Mars, Incorporated, 6885 Elm Street, McLean, Virginia 22101, United States; # Genome Center, University of California, Davis, California 95616, United States; ∇ Department of Biochemistry and Molecular Medicine, University of California, Davis, Sacramento, California 95616, United States

**Keywords:** rare sugars, d-tagatose, metabolic engineering

## Abstract

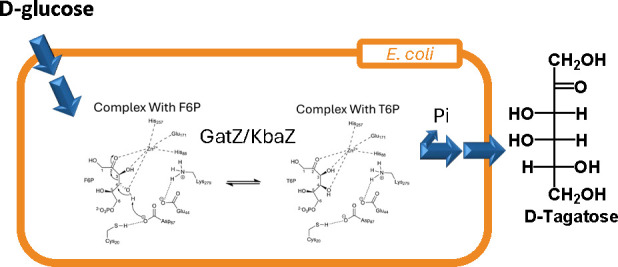

d-Tagatose, a rare low-calorie sweetener, is ideal for beverages due to its high solubility and low viscosity. Current enzymatic production methods from d-galactose or d-galactitol are limited by reaction reversibility, affecting the yield and purity. This study demonstrates that *Escherichia coli* harbors a thermodynamically favorable pathway for producing d-tagatose from d-glucose via phosphorylation–epimerization–dephosphorylation steps. GatZ and KbaZ, annotated as aldolase chaperones, exhibit C4 epimerization activity, converting d-fructose-6-phosphate to d-tagatose-6-phosphate. Structural analysis reveals active site differences between these enzymes and class II aldolases, indicating functional divergence. By exploiting the strains’ inability to metabolize d-tagatose, carbon starvation was applied to remove sugar byproducts. The engineered strains converted 45 g L^–1^d-glucose to d-tagatose, achieving a titer of 7.3 g L^–1^ and a productivity of 0.1 g L^–1^ h^–1^ under test tube conditions. This approach highlights *E. coli* as a promising host for efficient d-tagatose production.

## Introduction

Growing awareness regarding the health implication of eating habits has spurred a significant demand for healthier food options.^1^ In particular, there has been increased scrutiny regarding the role of dietary sugars in noncommunicable diseases.^2−4^ In response to this public health problem, the food industry is adopting artificial sweeteners as a means to lower sugar caloric content without compromising consumer satisfaction, evident in the projected growth of the sugar substitute market, which is expected to reach 29.9 billion USD by 2029.^5^

**Figure 1:**
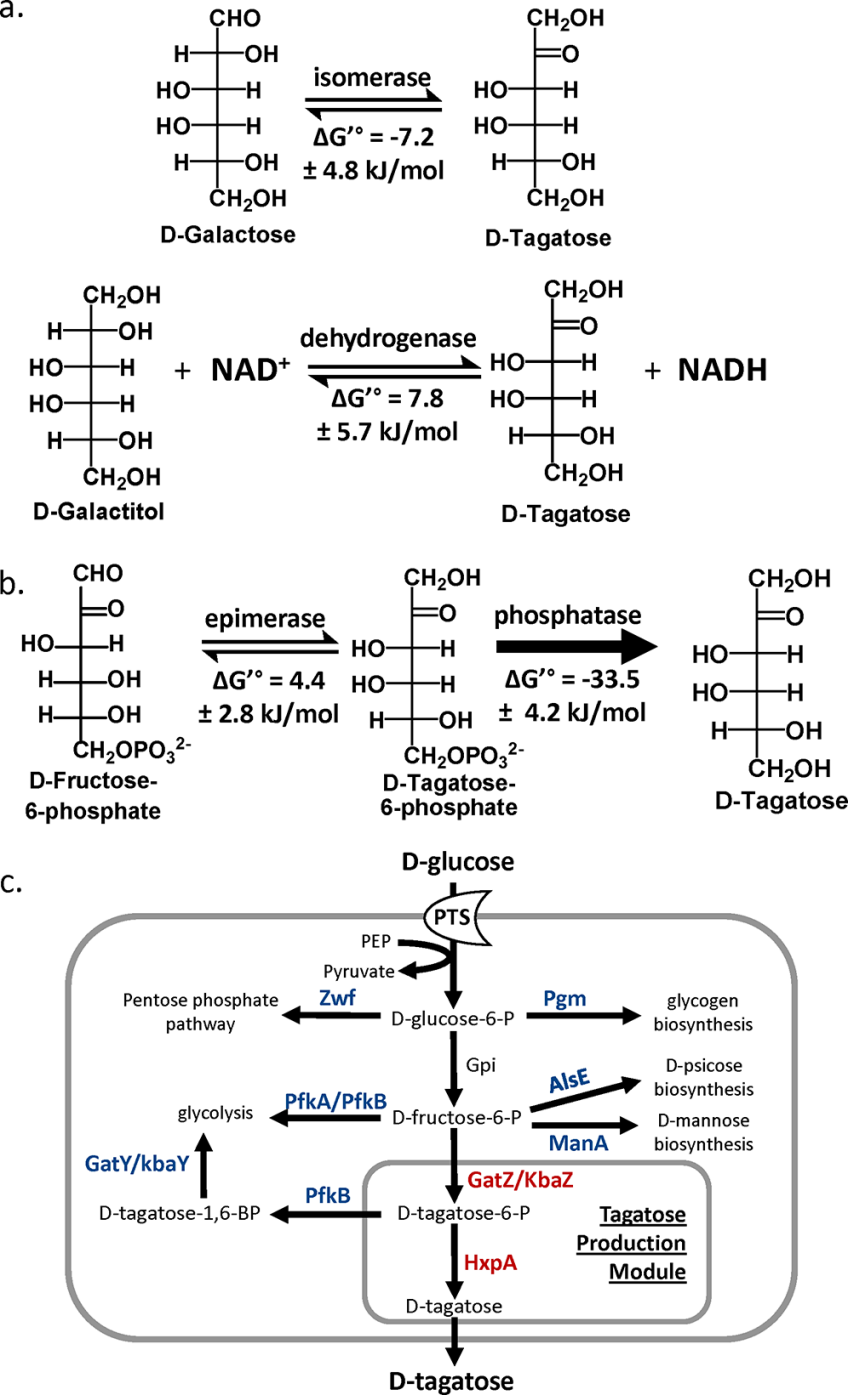
Strategies for the biosynthesis of d-tagatose. a) “Izumoring strategies” for d-tagatose production. b) Phosphorylation/dephosphorylation strategy for d-tagatose. c) The proposed biosynthetic production of d-tagatose in *E. coli*. Deleted steps are in blue. Additionally expressed steps are in red. PTS, the phosphotransferase system; GatZ, putative tagatose-1,6-bisphosphate aldolase 2 chaperone; KbaZ, putative tagatose-1,6-bisphosphate aldolase 1 chaperone; HxpA, hexitol phosphatase A; Zwf, NADP^+^-dependent glucose-6-phosphate dehydrogenase; Pgm, phosphoglucomutase; PfkA, 6-phosphofructokinase 1; PfkB, 6-phosphofructokinase 2; AlsE, d-allulose-6-phosphate 3-epimerase; ManA, mannose-6-phosphate isomerase; GatY, tagatose-1,6-bisphosphate aldolase 2; KbaY, tagatose-1,6-bisphosphate aldolase 1.

The current sweetener market is predominantly comprised of nonsugar substitutes, despite recent guidelines released by the World Health Organization advising against using nonsugar sweeteners for weight loss or preventing noncommunicable diseases.^6^ Rare sugars, characterized by their slight chemical structure variations compared to common sugars like glucose, are increasingly sought after as alternative sweeteners.^7^d-tagatose, a rare sugar and C4 epimer of fructose, has received Generally Recognized as Safe status and is 92% as sweet as sucrose.^8^d-tagatose is defined as a low-calorie sweetener, although there remains a debate regarding its calorific value, which ranges from 1.5 kcal g^–1^ to 3 kcal g^–1^.^7^d-tagatose has high solubility [58% (w/w) at 21 °C] and lower viscosity than sucrose which makes it an ideal sugar substitute in beverages.^9^ Moreover, its favorable flavor profile and desirable browning effects further enhance its suitability for use as a sugar substitute.^9^d-tagatose is reported to aid in weight loss and exhibits additional beneficial effects such as antiplaque, noncariogenic, antihalitosis, prebiotic, and antibiofilm properties.^9^

d-tagatose is found in minute concentration in sterilized powdered milk, hot cocoa, cheese, yogurt, and other dairy products.^10^ However, the extraction of d-tagatose from natural sources is not economically feasible due to complex purification steps and low yield.^9^ Synthetic methods for d-tagatose production also suffer from similar disadvantages along with the additional use of acids, bases, and catalysts.^10^ A potential solution to this problem is the “Izumoring” strategy which makes use of enzymes and serves as a framework for the interconversion of aldohexoses, ketohexoses, and hexitols.^11^ In this regard, two such enzymes have been extensively investigated (Figure 1a): 1) l-arabinose isomerase (EC 5.3.1.4), which interconverts d-galactose and d-tagatose, and 2) galactitol 2-dehydrogenase (EC 1.1.1.16), which interconverts galactitol and d-tagatose.^8,12^ However, these methods encounter challenges such as the requirement for purified enzymes, cofactors like NAD^+^, low thermal stability, or a lack of thermodynamic driving force, resulting in low yields and high cost of production.^13,14^ To address these challenges, recent research encapsulated l-arabinose isomerase in *Lactobacillus plantarum*, achieving high yields of d-tagatose. However, this method relied on high-cost d-galactose as the starting material, which is needed to be separated from d-tagatose postproduction, further elevating costs.^15^ Alternatively, microbial production was applied to convert lactose to d-tagatose in *Saccharomyces cerevisiae*, but this method utilizes only d-galactose from lactose and d-glucose is used to maintain cell growth; thus the yield was low.^16^ Consequently, the final media contained galactose, galactitol, and d-tagatose, resulting in higher separation and purification costs.^16^

While d-fructose holds potential as a starting material for d-tagatose production, a notable gap exists in nature: the absence of a C4 epimerase capable of directly converting d-fructose to d-tagatose.^17−19^ A C4-epimerization activity of the tagaturonate-fructuronate epimerase from *Thermotoga petrophila* was identified.^19^ This enzyme converted 700 g L^–^^1^ of fructose into 213 g L^–1^ of tagatose within 2 h.^19^ Further optimization through directed evolution improved its thermal stability and activity, demonstrating its potential for d-tagatose production.^20^

Recently, a phosphorylation and dephosphorylation strategy has surfaced as a promising route for rare sugar production from glucose, presenting an economically advantageous feedstock.^21,22^ This pathway involves a pivotal C4 epimerization reaction, transforming d-fructose-6-phosphate (F6P) into d-tagatose-6-phosphate (T6P). Subsequent dephosphorylation of T6P provides a substantial thermodynamic driving force, facilitating efficient d-tagatose production. Utilizing this pathway, whole-cell biocatalysts have been developed, yielding 3.38 g/L of d-tagatose from 10 g/L of maltodextrin.^21^ However, this approach requires high cell density and operates with a relatively low substrate loading, posing significant challenges for industrial scalability.^21^ Additionally, incomplete conversion leaves residual glucose and fructose in the solution, highlighting inefficiencies in the process and the need for further optimization.^21^

In this study, we discovered that *E. coli* has all of the required enzymes to convert d-glucose to d-tagatose (Figure 1b). d-Glucose is converted to F6P via glycolysis, and the epimerization of F6P to T6P is accomplished by native enzymes: GatZ, a putative tagatose-1,6-bisphosphate aldolase 2 chaperone,^23^ or KbaZ, a putative tagatose-1,6-bisphosphate aldolase 1 chaperone.^24^ Although these enzymes are annotated as aldolase chaperones,^25^ this study demonstrates the C4 epimerization activity of these enzymes in *E. coli*, enabling the conversion of F6P to T6P. Structural analyses of GatZ and KbaZ highlight critical differences in their active sites compared to class II fructose-1,6-bisphosphate aldolases (FBAs), including substitutions of key residues involved in metal coordination and substrate stabilization. These conserved differences across the two enzyme families provide insights into the functional divergence, enabling the unique activity of GatZ and KbaZ. Moreover, the dephosphorylation of T6P can be achieved by hexitol-phosphatase A (HxpA). Production of d-tagatose from d-glucose in *E. coli* was achieved only with the native genes of *E. coli*. d-tagatose production was further improved by additionally expressing the pathway genes and removing competing pathways, including the pentose phosphate pathway, glycogen biosynthesis, glycolysis, d-psicose production pathway, and d-mannose degradation pathway.

## Materials and Methods

### Reagents

All enzymes involved in the molecular cloning experiments were purchased from New England Biolabs (NEB). All synthetic oligonucleotides were synthesized by Integrated DNA Technologies. Sanger Sequencing was provided by Genewiz. d-Mannitol and d-Tagatose were purchased from the Tokyo Chemical Industry. d-Psicose and d-Mannose were purchased from Sigma-Aldrich. d-Glucose and d-Fructose were purchased from Fisher Scientific.

### Strains and Plasmids

All strains and plasmids used in this study are listed in Tables 1 and 2, respectively. All oligonucleotides are given in Table S1. Plasmids for d-tagatose production were constructed using sequence and ligation-independent cloning (SLIC).^26^ The constructed plasmids were verified via sequencing. A guide to the construction of plasmids used in this study is given in Table S2.

**Table 1:** Strains Used in This Study

Strain	Genotype	Source
MG1655	F-lambda-*ilvG*-*rfb*-50 *rph*-1	(62)
AL1050	MG1655, but attB:*lacI*^q^*tetR* spec^R^	(63)
AL3755	AL1050, but Δ*pfkA*	This study
AL4240	AL3755, but Δ*zwf*	This study
AL4290	AL4240, but Δ*manA*	This study
AL4314	AL4240, but Δ*alsE*	This study
AL4315	AL4290, but Δ*alsE*	This study
AL4330	AL4315, but Δ*pgm*	This study
AL4333	AL4330, but Δ*gatY*	This study
AL4424	AL4240, but Δ*gatZ*	This study
AL4425	AL4240, but Δ*kbaZ*	This study
AL4493	AL4330, but Δ*kbaY*	This study
AL4517	AL4240, but Δ*kbaY*	This study
AL4532	AL4240, but Δ*gatY*	This study
AL4534	AL4493, but Δ*gatY*	This study

**Table 2:** Plasmids Used in This Study

Plasmid	Description	Source
pAL1950	pTargetF-*pfkA*, amp^R^, ColE1	(22)
pAL1958	pTargetF-*zwf*, amp^R^, ColE1	(22)
pAL2038	pTargetF-*pgm*, ampR, ColE1	(22)
pAL2178	pTargetF-*manA*, ampR, ColE1	(22)
pAL2233	pTargetF-*alsE*, ampR, ColE1	(22)
pAL2475	pTargetF-*gatZ*, amp^R^, ColE1	This study
pAL2480	*P*_LlacO1_: *gatZ*, amp^R^, ColE1	This study
pAL2483	pTargetF-*kbaZ*, amp^R^, ColE1	This study
pAL2490	*P*_LlacO1_: *gatZ-hxpA*, amp^R^, ColE1	This study
pAL2491	*P*_LlacO1_: *gatZ-hxpB*, amp^R^, ColE1	This study
pAL2492	*P*_LlacO1_: *gatZ-ybiV*, amp^R^, ColE1	This study
pAL2493	*P*_LlacO1_: *gatZ-yidA*, amp^R^, ColE1	This study
pAL2494	*P*_LlacO1_: *gatZ-yigL*, amp^R^, ColE1	This study
pAL2495	*P*_LlacO1_: *gatZ-yihX*, amp^R^, ColE1	This study
pAL2496	*P*_LlacO1_: *gatZ-yqaB*, amp^R^, ColE1	This study
pAL2497	*P*_LlacO1_: *kbaZ-hxpA*, amp^R^, ColE1	This study
pAL2189	pTargetF-*kbaY*, amp^R^, ColE1	This study
pAL2190	pTargetF-*gatY*, amp^R^, ColE1	This study
pAL2568	pTargetF-*fbaA*, ampR, ColE1	This study
pAL2574	*P*_LlacO1_: gatZ-hxpA* (1G > A), amp^R^, ColE1	This study
pAL2575	*P*_LlacO1_: kbaZ-hxpA* (1G > A), amp^R^, ColE1	This study
pAL2586	*P*_LlacO1_: *fbaA-hxpA*, amp^R^, ColE1	This study
pAL2587	*P*_LlacO1_: *gatY-hxpA*, amp^R^, ColE1	This study
pAL2588	*P*_LlacO1_: *kbaY-hxpA*, amp^R^, ColE1	This study
pAL2606	*P*_*gadB*_: *gatZ-hxpA* (1G > A), amp^R^, ColE1	This study
pAL2607	*P*_*gadB*_: *kbaZ-hxpA* (1G > A), amp^R^, ColE1	This study
pCas	*P*_*cas*_:*cas9 P*_*araB*_:red lacI^q^*P*_trc_:sgRNA pMB1 repA101(Ts) kan^R^	addgene #62225
pTargetF	PJ23119:sgRNA-pMB1, spec^R^, pMB1	addgene #62226

Genome modifications such as gene deletion and gene insertion were constructed using CRISPR-Cas9-mediated homologous recombination.^27^ Linear DNA repair fragments for gene deletions and insertions were constructed by amplifying genomic or plasmid DNA via PCR assembly.^27^ Plasmids encoding sgRNA for CRISPR-Cas9-mediated homologous recombination were constructed using Q5 site-directed mutagenesis (New England Biolabs) using pTargetF plasmid (Addgene no. 62226) as a template. All genomic modifications were verified via PCR and sequencing. A guide to the CRISPR-Cas9-mediated gene modifications used in this study is detailed in Table S3.

### Culturing Conditions

Overnight cultures were grown at 37 °C in 3 mL of Luria–Bertani (LB) medium with appropriate antibiotics. Antibiotic concentrations were as follows: spectinomycin (50 μg mL^–1^), ampicillin (200 μg mL^–1^), and kanamycin (50 μg mL^–1^). M9 minimal media consist of 33.7 mM Na_2_HPO_4_, 22 mM KH_2_PO_4_, 8.6 mM NaCl, 9.4 mM NH_4_Cl, 2 mM MgSO_4_, 0.1 mM CaCl_2_, A5 trace metals mix (2.86 mg L^–1^ H_3_BO_3_, 1.81 mg L^–1^ MnCl_2_·4H_2_O, 0.079 mg L^–1^ CuSO_4_·5H_2_O, 49.4 μg L^–1^ Co(NO_3_)_2_·6H_2_O), varying concentrations of glucose, and appropriate antibiotics. M9P media for the d-tagatose production consist of M9 minimal media supplemented with 5 g L^–1^ of yeast extract and appropriate antibiotics. Inducer concentrations are as follows: isopropyl-β-d-1-thiogalactopyranoside (IPTG) (1 mM). The optical density at 600 nm (OD_600_) was measured with a Synergy H1 hybrid plate reader (BioTek Instruments, Inc.).

### d-Tagatose Production

For regular cell density production experiments, overnight cultures were inoculated at 1% concentration into 3 mL of M9P media. Cells were grown at 37 °C until the OD_600_ described, then induced with IPTG if necessary, and grown at 30 °C for 24 h.

For high cell density production experiments in M9P media, overnight cultures were inoculated at 1% concentration into 150 mL of M9P media. Cells were grown at 37 °C until OD_600_ 0.4–0.6. Cultures were then induced with IPTG if necessary and grown for another 30 min. Cultures were centrifuged at 2,200 *g* for 5 min and resuspended in M9P media with IPTG if necessary to a target OD_600_. Cultures were grown at 30 °C.

### Structural Analysis

To examine the conservation between the GatZ/KbaZ family and the fructose 1,6-bisphosphate aldolase (Fba) family, we performed a sequence-based analysis. First, protein sequences corresponding to each family were retrieved from UniProt.^28^ The GatZ/KbaZ family was anchored to the GatZ sequence (UniProt Accession: P0C8J8), and the Fba family was anchored to the Fba sequence (UniProt Accession: A8B2U2).

To examine structural similarity, we employed the Foldseek web application^29^ by searching against *E. coli* GatZ (UniProt/AlphaFoldDB: P0C8J8). Structural similarity was assessed based on the calculated TM-score provided by Foldseek, which utilizes the standard TM-align algorithm.^30^ After applying a PDB100 filter, we manually examined the resulting entries to identify structurally similar FBA crystal structures featuring ligands in catalytically relevant poses. The collected sequences were aligned using Clustal Omega, a widely used multiple sequence alignment tool, to ensure reliable and accurate alignment across all family members.^31^ Conservation analyses were subsequently conducted using JalView, an integrated platform for sequence visualization and analysis.^32^ JalView’s conservation scoring feature was applied to calculate the degree of conservation across the alignment, with GatZ and Fba sequences serving as anchors for their respective families. These anchors ensured consistent reference points for assessing family-level conservation.

The Rosetta Molecular Suite was used to dock the ligand fructose-6-phosphate (F6P), into the epimerases GatZ, KbaZ, and the ligand tagatose-6-phosphate (T6P) into the phosphatase HxpA.^33^ F6P AM1-BCC partial charges were assigned using the Antechamber suite from AMBER.^34^ For the initial protein structures, we used AlphaFold version 2.3.0.^35^ The AlphaFold-predicted models were then prepared for docking by using Rosetta Relax.^36^ The ligand was then placed in the active site. Rosetta GALigand Dock was then used to dock F6P conformers into each enzyme.^37^ Distance and angle constraints were integrated to maintain the d-fructose-6-phosphate and zinc ion in a catalytically competent geometry for aldolase-based epimerization. A total of 2,500 simulations were run for each enzyme, and the top 10 best-scoring outputs sorted by constraint score, protein–ligand interface energy, and total system energy score were selected for analysis.

Details of the input files used, including constraints, RosettaScripts XML, and ligand parameters, can be found in the Supporting Information. Detailed instructions with sample data can be found on our GitHub at https://github.com/siegel-lab-ucd/D-tagatose-production.

### HPLC Analysis

Analysis of d-tagatose, d-glucose, d-mannose, d-psicose, d-mannitol, and d-fructose concentrations was performed using HPLC (Shimadzu) equipped with a refractive index detector (RID) 10 A and Rezex RCU-USP sugar alcohol column (Phenomenex). The mobile phase was comprised of 100% Milli-Q water. Samples were run with an injection volume of 1 μL at a flow rate of 0.5 mL min^–1^ for 7.5 min, with the column oven at 83 °C and RID cell temperature of 40 °C.

To prepare samples for HPLC analysis, 300 μL of culture was centrifuged at 17,000 *g* for 5 min. Supernatants were applied to a 0.2 μm PVDF hydrophilic membrane 96-well filter plate and centrifuged at 2,000 rpm for 2 min into a polystyrene 96 well.

## Results and Discussion

### Natural d-Tagatose Production Capability of *E. coli*

**Figure 2:**
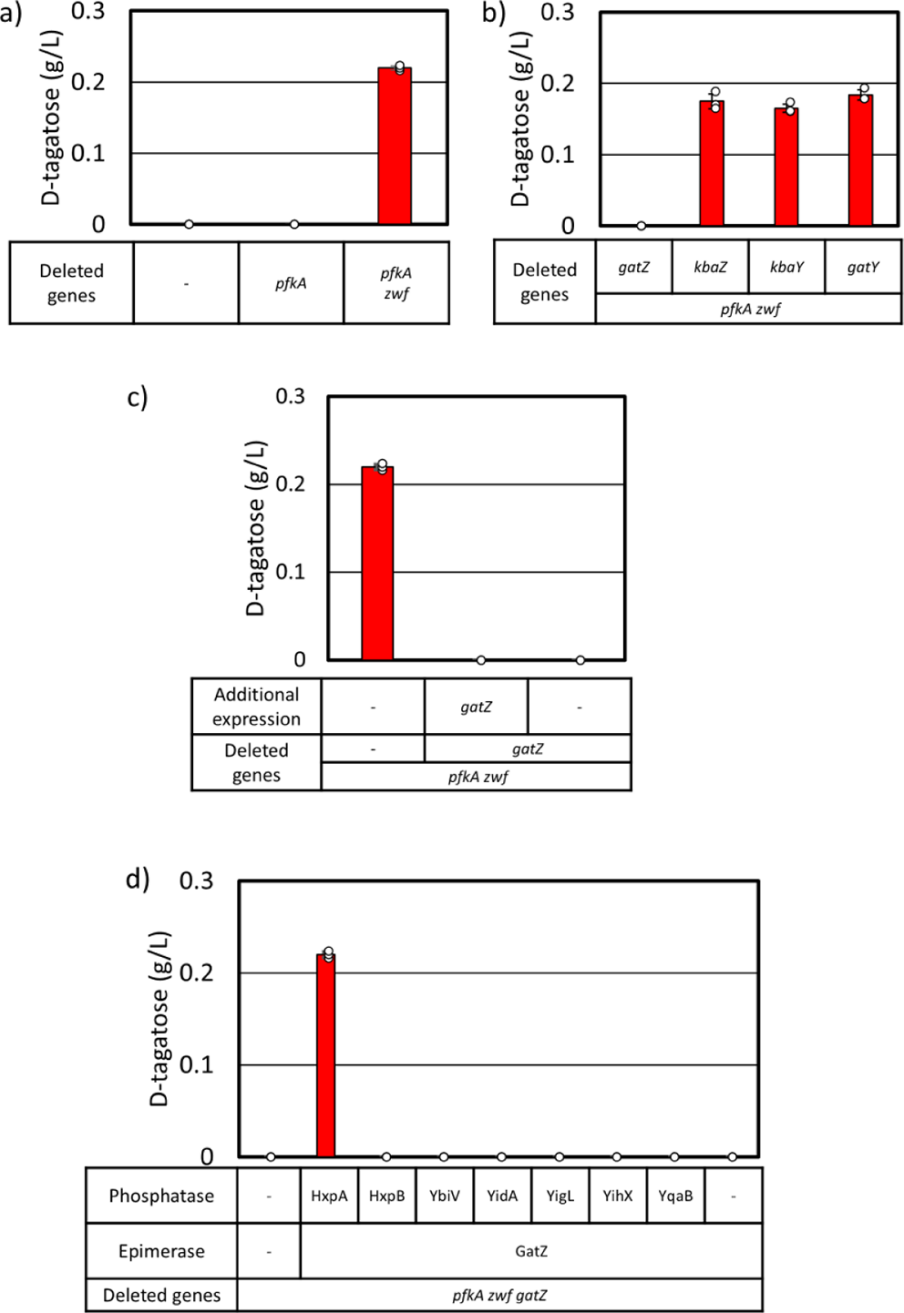
d-tagatose production capability of*E. coli*. Cells were grown in M9P media with 10 g L^–1^ glucose at 37 °C to OD_600_ ∼ 0.4, then grown at 30 °C for 24 h. At OD_600_ ∼ 0.4, 1 mM IPTG was added (c, d). a) d-tagatose production in AL1050 (MG1655 + *lacI*^q^*tetR* spec^R^), AL3755 (AL1050 with Δ*pfkA*), and AL4240 (AL1050 with Δ*pfkA* Δ*zwf*) (Table 1). b) Effect of each epimerase gene deletion on d-tagatose production in AL4240. c) d-tagatose production in AL4424 (AL1050 with Δ*pfkA* Δ*zwf* Δ*gatZ*) with and without additional expression of *gatZ*. (Table 1 and 2). d) Effect of additional expression of each phosphatase gene and *gatZ* on d-tagatose production in AL4424 (AL1050 with Δ*pfkA* Δ*zwf* Δ*gatZ*). Error bars indicate s.d. (*n* = 3 biological replicates).

The natural ability of *E. coli* to produce d-tagatose from d-glucose was examined. The production strain, derived from MG1655 (Table 1), did not produce d-tagatose under the production conditions (Figure 2a). To accumulate a critical intermediate, F6P, the *pfkA* gene encoding for phosphofructokinase A was deleted, but the Δ*pfkA* strain did not produce d-tagatose (Figure 2a). The *zwf* gene encoding glucose-6-phosphate dehydrogenase was deleted to further increase the F6P pool. The Δ*pfkA* Δ*zwf* strain (AL4240, Table 1) produced 0.21 g L^–1^d-tagatose in 24 h (Figure 2a) indicating that *E. coli* naturally has all required enzymes to produce d-tagatose from d-glucose.

### Elucidating the d-Tagatose Production Pathway in *E. coli*

While *E. coli* has been used to produce d-tagatose, these efforts largely relied on heterologous enzymes.^21,38,39^ We postulated that the natural d-tagatose pathway involves the conversion of d-glucose to F6P through glycolysis, followed by the action of an epimerase to convert F6P into T6P. Subsequently, dephosphorylation of T6P by a phosphatase yields d-tagatose (Figure 1b).

We identified five endogenous enzymes as candidates for possessing C4 epimerase activity to convert F6P into T6P. First, it has been shown that FBA encoded by *fbaA* exhibits C4 epimerase activity *in vitro.*(18,40) Next, although GatZ is annotated as a putative tagatose-1,6-bisphosphate aldolase 2 chaperone, it was used to facilitate growth of *Agrobacterium tumefaciens* on galactitol, suggesting GatZ can convert T6P to F6P, which is the reverse reaction used in this study.^41^ Additionally, we found KbaZ annotated as a tagatose-1,6-bisphosphate aldolase 2 chaperone has high structural similarity with GatZ with a TM-Score of 0.9707.^30^ Finally, the enzymes GatY (tagatose-1,6-bisphosphate aldolase 2) and KbaY (tagatose-1,6-bisphosphate aldolase 1) have high structural similarity with FbaA with TM-scores of 0.92352 and 0.92851, respectively.^25,30^

In order to elucidate the C4 epimerase involved in the production of d-tagatose, each candidate gene was deleted in AL4240 (Δ*pfkA* Δ*zwf*, Table 1). The *fbaA* gene could not be deleted as it is essential.^42^ Tagatose production was abolished only with the deletion of *gatZ* (Figure 2b), suggesting GatZ converts F6P to T6P in *E. coli*. However, the expression of *gatZ* under the IPTG-inducible promoter *P*_LlacO1_,^43^ from an expression plasmid in AL4424 (Δ*pfkA* Δ*zwf* Δ*gatZ*, Table 1) did not compensate d-tagatose production (Figure 2c). It has been demonstrated that the expression of *gatZ* enhances the activity of tagatose-1,6-bisphosphate aldolases,^25^ suggesting a potential diversion of T6P away from d-tagatose production toward glycolysis. We hypothesized that additional expression of T6P phosphatase is required to redirect the carbon flux to d-tagatose production. We selected promiscuous phosphatases^44^ and conducted screening by expressing hexitol phosphatase B (HxpB), sugar phosphatase YbiV, sugar phosphatase YidA, hexitol phosphatase A (HxpA), α-d-glucose-1-phosphate phosphatase YihX, phosphosugar phosphatase YigL, or fructose-1-phosphate phosphatase YqaB along with GatZ in AL4424 (Δ*pfkA* Δ*zwf* Δ*gatZ*, Table 1). Coexpression of GatZ and hexitol phosphatase A (HxpA) restored d-tagatose production in AL4424 (Figure 2d).

**Figure 3:**
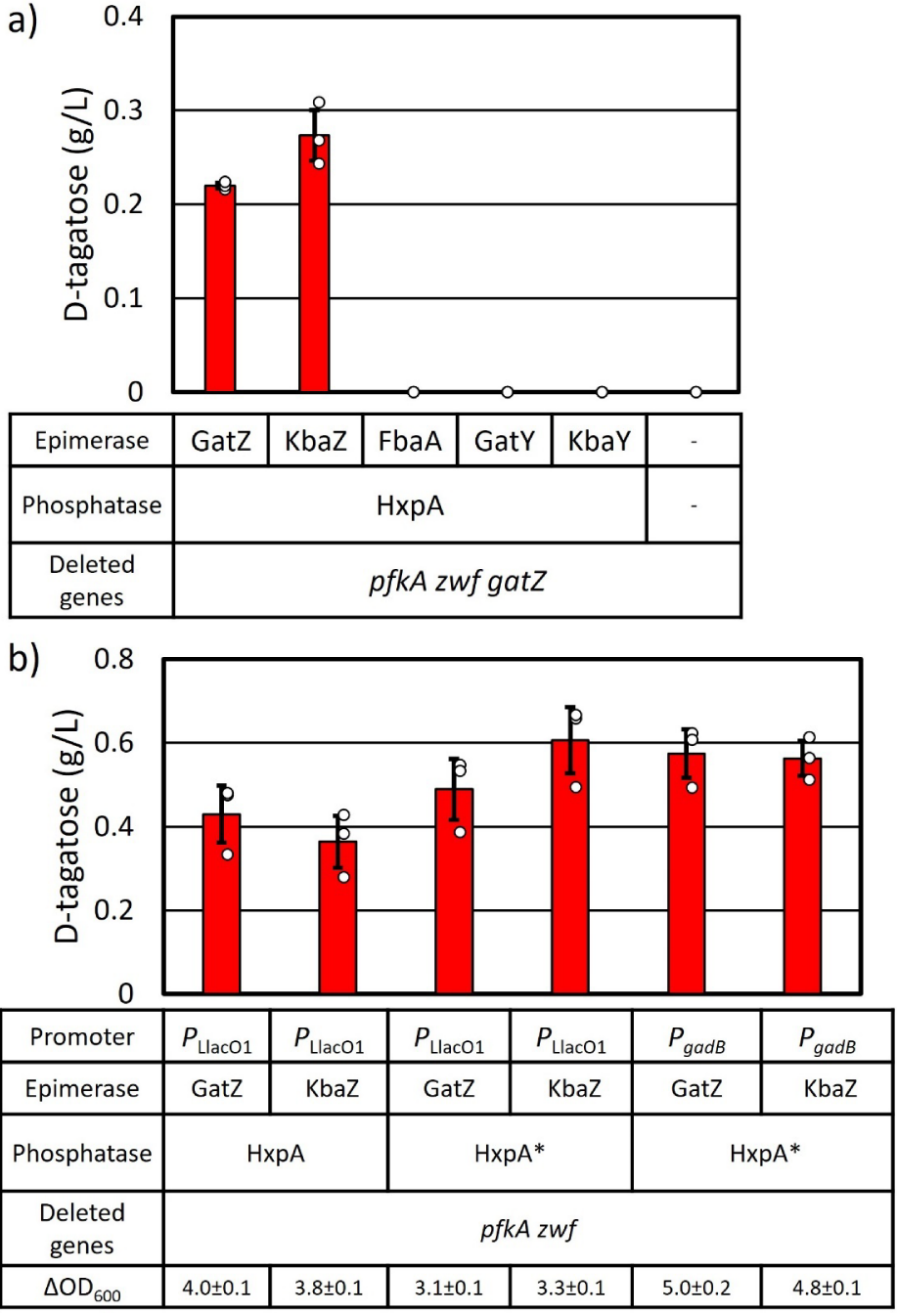
Modulating expression of genes for d-tagatose production. Cells were grown in M9P media with 10 g L^–1^ glucose at 37 °C to OD_600_ ∼ 0.4, then grown at 30 °C for 24 h. At OD_600_ ∼ 0.4, 1 mM IPTG was added when required. a) Each candidate epimerase gene was expressed along with *hxpA* under *P*_LlacO1_ promoter (Table 2) in AL4424 (AL1050 with Δ*pfkA* Δ*zwf* Δ*gatZ*, Table 1). b) Either *gatZ* or *kbaZ* and *hxpA* were expressed under *P*_LlacO1_ or *P*_gadB_. The start codon of *hxpA* was changed from GTG to ATG (hxpA*). Errors indicate s.d. (*n* = 3 biological replicates).

The other C4 epimerase candidate genes may not be expressed under our culture conditions. Therefore, each epimerase candidate was expressed along with *hxpA* from an expression plasmid in AL4424 (Δ*pfkA* Δ*zwf* Δ*gatZ*, Table 1). The additional expression of *kbaZ* restored d-tagatose production, but that of *fbaA*, *kbaY*, or *gatY* did not (Figure 3a). *gatZ* and *kbaZ* were used for further modifications.

### Analysis of the Structural Similarity of the C4 Epimerase

The search for a C4-epimerase to facilitate the conversion of d-fructose to d-tagatose has been an important effort in the sugar industry.^19^ A C4-epimerization capability of the tagaturonate-fructuronate epimerase from *Thermotoga petrophila* was identified.^19^ However, this reaction is thermodynamically unfavorable, leading to a mixture of d-fructose and d-tagatose. An enzyme capable of converting F6P to T6P from *Agrobacterium tumefaciens* C58 was identified.^45^ This finding enabled a phosphorylation-dephosphorylation pathway for d-tagatose production.^21^ Another C4 epimerase was identified in *Sinorhizobium meliloti* which showed structural similarity to GatZ of *E. coli*.^41^

Although the GatZ/KbaZ family of proteins has been studied since 2002,^25^ the reaction mechanisms have not been characterized well. Initially, these proteins were classified as fructose 1,6-bisphosphate aldolase chaperones due to their conservation to known fructose 1,6-bisphosphate aldolases, despite lacking aldolase activity under the conditions used in one study.^25^ More recently, GatZ has been shown to exhibit epimerase activity, catalyzing the epimerization of T6P into F6P^41^, as well as the reverse reaction from F6P to T6P.^21,46^

**Figure 4:**
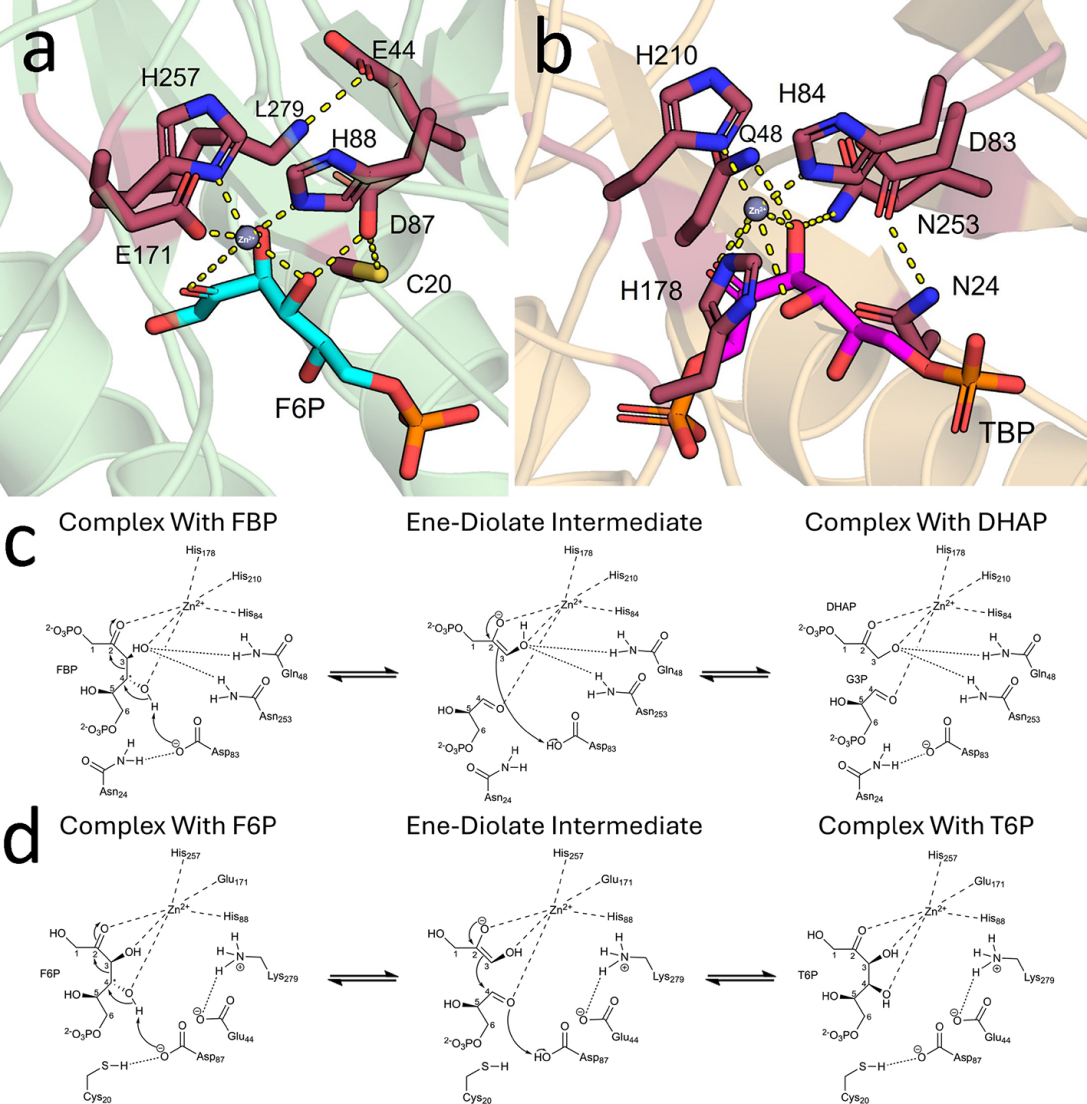
Structure analysis of GatZ. a) Predicted substrate-binding mode of the AlphaFold structure of GatZ with F6P. b) Substrate-binding model of FBA (PDB: 3GAY) with d-tagatose-1,6-diphosphate. Zn^2+^ ions are shown as gray spheres in the structural models in (a) and (b). c) Reaction mechanism of FBA catalyzing the conversion of fructose-1,6-bisphosphate to dihydroxyacetone phosphate (DHAP) and glyceraldehyde-3-phosphate (G3P). d) Reaction mechanism of GatZ catalyzing the conversion of F6P to T6P.

AlphaFold2^35^ was used to predict the three-dimensional structures of GatZ and KbaZ as part of an investigation into their interactions with F6P. Rosetta^33^ was used to model and simulate their interactions (Figure 4). Epimerization at the C4 position is predicted to occur through a zinc-dependent retro-aldol/aldol mechanism, similar to that of known C4 epimerases such as l-ribulose-5-phosphate 4-epimerase (AraD),^47^ fructose-bisphosphate aldolase class II (FbaA),^18^ and other C4 epimerases.^47^ In the case of GatZ, the active site contains a Zn^2+^ cofactor coordinated by two conserved histidine residues (His88 and His257) and a glutamic acid residue (Glu171) (Figure 4a,d). Additionally, an aspartate residue (Asp87) is present (Figure 4a,d). The reaction is predicted to be initiated when Asp87 deprotonates the C4 hydroxyl group of F6P, leading to C3–C4 bond cleavage to form dihydroxyacetone phosphate and glyceraldehyde intermediates. Subsequently, an aldol condensation reforms the C3–C4 bond with inverted stereochemistry at C4, producing tagatose 6-phosphate (Figure 4d).

Under the specific conditions described in one study, GatZ was unable to perform the aldolase reaction.^25^ Despite this, GatZ shares an evolutionary relationship with common class II FBAs.^25,29,47^ Additionally, aldol cleavage by class II fructose-1,6-bisphosphate aldolases proceeds through a similar mechanism (Figure 4c). Given the mechanistic similarities between an aldol cleavage and an aldol/retro-aldol epimerization, we sought to understand why this limitation exists. We compared the predicted catalytic pose of GatZ (Figure 4a) with the crystal structure of a known fructose 1,6-bisphosphate aldolase (FBA) (PDB: 3GAY) complexed with tagatose 1,6-bisphosphate (TBP) (Figure 4b).^48^ Due to the limited availability of experimentally determined crystal structures of FBA with its native substrate fructose 1,6-bisphosphate (FBP), we opted to use 3GAY complexed with TBP. The structural similarities between TBP and FBP result in them adopting virtually identical binding poses within the enzyme’s active site. Therefore, comparing the predicted catalytic pose of GatZ with the TBP-bound crystal structure is appropriate, allowing us to note several key differences.

Due to their high structural similarity, GatZ was chosen to represent the GatZ/KbaZ family within the main body for clarification. In FBA, metal coordination is carried out by three conserved histidine residues (His84, His178, and His210; Figure 4b). In contrast, in GatZ, metal coordination is carried out by two conserved histidine residues and a glutamic acid residue (His88, His257, and Glu171) (Figure 4a). Additionally in FBA, C3-OH is stabilized by hydrogen bonding carried out by Asp253 and Gln48. Hydrogen bonding stabilizing C3-OH has been lost, and the corresponding residues have been replaced with a lysine (Lys279) and a glutamic acid (Glu44) in GatZ. Finally, the FBA stabilizes the catalytic aspartic acid through an asparagine (Asn24), while in GatZ it has been replaced by a cysteine (Cys20). To confirm that these differences are unique to these families, sequences of fructose-1,6-bisphosphate (FBP) aldolase class II FbaA (IPR050246) and the GatZ/KbaZ-like family (IPR012062) were aligned using EMBL-EBI’s Clustal Omega.^31^ These differences are highly conserved across both families (Table S4) and are key differentiating factors between the two families. The observed differences between these two active sites do not clearly explain their differential preference toward one reaction or the other. Our computational analysis has generated a testable hypothesis for the GatZ/KbaZ epimerization mechanism. To evaluate the molecular determinants underlying their unique catalytic properties, further experimental validation is essential. Future studies employing techniques such as site-directed mutagenesis of key active site residues and molecular dynamics simulations will be critical for providing evidence that supports our proposed mechanism and for elucidating the evolutionary adaptations responsible for their functional divergence from canonical FBAs.^49−52^

### Modulating the Expression of the d-Tagatose Production Pathway Genes

The native *hxpA* gene has a GTG start codon. In order to increase translation efficiency of *hxpA*, the start codon was changed to ATG (hxpA*).^53^ This modification improved d-tagatose titers to 0.48 g L^–1^ with the additional expression of *gatZ* and 0.60 g L^–1^ with the additional expression of *kabZ* (Figure 3b). The *P*_LlacO1_ promoter was replaced with the *P*_gadB_ promoter which is a stationary phase promoter and ∼100 times stronger than *P*_LlacO1_.^22^ The d-tagatose productivity with *P*_*gadB*_ was similar to that with *P*_LlacO1_ (Figure 3b).

### Removing Competing Pathways

**Figure 5:**
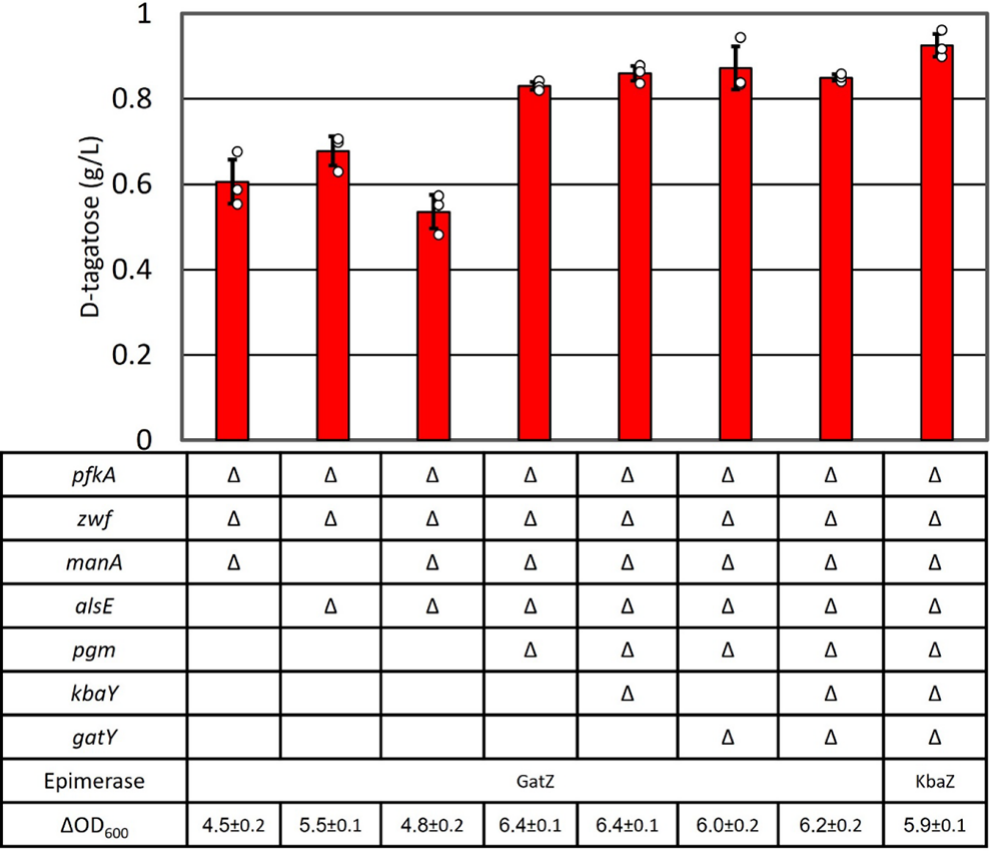
The effect of gene deletions on d-tagatose production. Cells were grown in M9P media with 10 g L^–1^ glucose to OD_600_ ∼ 0.4 at 37 °C, then grown at 30 °C for 24 h. Errors indicate s.d. (*n* = 3 biological replicates).

AL4240 (Δ*pfkA* Δ*zwf*, Table 1) with production plasmid produced 0.8 g L^–1^d-mannose and 1.1 g L^–1^d-psicose along with d-tagatose (Figure S1). The *manA* gene encodes mannose-6-phosphate isomerase, which interconverts F6P and mannose-6-phosphate,^54^ diverting carbon flux toward d-mannose production. The *alsE* gene encodes d-allulose 6-phosphate 3-epimerase, which interconverts F6P and d-psicose-6-phosphate, is responsible for the production of d-psicose.^22^ The deletion of *manA* in AL4240 (Δ*pfkA* Δ*zwf*, Table 1) resulted in the creation of AL4290 (Δ*pfkA* Δ*zwf* Δ*manA*, Table 1). This deletion improved d-tagatose production to 0.6 g L^–1^ and eliminated d-mannose production (Figures 5, S1). Similarly, the deletion of *alsE* in AL4240 (Δ*pfkA* Δ*zwf*, Table 1) resulted in AL4314 (Δ*pfkA* Δ*zwf* Δ*alsE*, Table 1). This deletion improved d-tagatose production to 0.67 g L^–1^ and eliminated d-psicose production (Figures 5, S1). Both of these genes were deleted in AL4240 (Δ*pfkA* Δ*zwf*, Table 1), generating AL4315 (Δ*pfkA* Δ*zwf* Δ*alsE* Δ*manA*, Table 1). AL4315 (Δ*pfkA* Δ*zwf* Δ*alsE* Δ*manA*, Table 1) with the production plasmid produced 0.53 g L^–1^d-tagatose (Figure 5) and did not produce d-psicose and d-mannose (Figure S1).

*E. coli* can store excess glucose in the form of glycogen.^55^ The *pgm* gene encoding phosphoglucomutase converts G6P to glucose-1-phosphate.^56^ The *pgm* gene was deleted in AL4315 (Δ*pfkA* Δ*zwf* Δ*alsE* Δ*manA*, Table 1), generating AL4330 (Δ*pfkA* Δ*zwf* Δ*alsE* Δ*manA* Δ*pgm*, Table 1). AL4330 with the production plasmid produced 0.82 g of L^–1^d-tagatose (Figure 5).

T6P can be converted to d-tagatose 1,6-bisphosphate by tagatose-6-phoshate kinase (PfkB) that is subsequently converted to glycerone phosphate and d-glyceraldehyde 3-phoshate aldolase (GatY and KbaY).^25^ The *pfkB* gene cannot be deleted as the *pfkA* gene was deleted in the strains. It has been shown that overexpression of *gatZ* enhances tagatose-1,6-bisphosphate aldolase activity in *E. coli*.^25^ Activated tagatose-1,6-bisphosphate aldolase activity would divert carbon flux away from d-tagatose production and toward glycolysis. The *kbaY* gene was deleted in AL4330 (Δ*pfkA* Δ*zwf* Δ*alsE* Δ*manA* Δ*pgm*, Table 1), generating AL4493 (Δ*pfkA* Δ*zwf* Δ*alsE* Δ*manA* Δ*pgm* Δ*kbaY,*Table 1). Similarly, *gatY* was deleted in AL4330 (Δ*pfkA* Δ*zwf* Δ*alsE* Δ*manA* Δ*pgm*, Table 1), generating AL4533 (Δ*pfkA* Δ*zwf* Δ*alsE* Δ*manA* Δ*pgm* Δ*gatY,*Table 1). Both *gatY* and *kbaY* were deleted in AL4330 (Δ*pfkA* Δ*zwf* Δ*alsE* Δ*manA* Δ*pgm*, Table 1), generating AL4534 (Δ*pfkA* Δ*zwf* Δ*alsE* Δ*manA* Δ*pgm* Δ*gatY* Δ*kbaY,*Table 1). However, these deletions did not improve d-tagatose production (Figure 5).

### d-Tagatose Production Under High Culture Density Conditions

**Figure 6:**
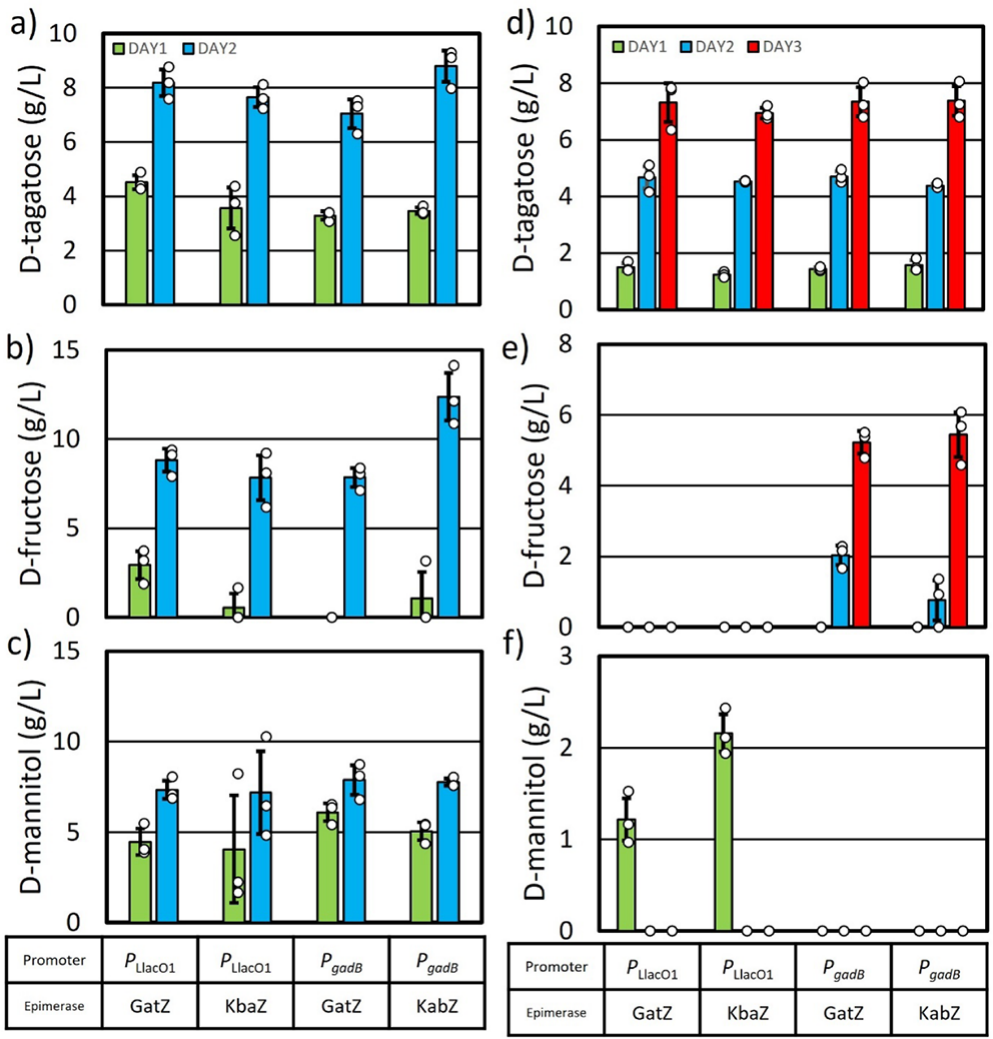
High cell density d-tagatose production. Cultures were grown in M9P media with 40 g L^–1^ (a–c) and 15 g L^–1^ (d–f) glucose concentration at 37 °C until an OD_600_ of ∼0.4–0.6. Induced with 1 mM IPTG if necessary and grown for a further 30 min. Cultures were then spun down and resuspended in M9P media with 40 g L^–1^ (a–c) and 15 g L^–1^ (d–f) glucose, induced with 1 mM IPTG if necessary, to an OD_600_ of ∼10 and grown at 30 °C. Each day M9P media with 40 g L^–1^ (a–c) and 15 g L^–1^ (d–f) glucose was added to the production media. (a and d) d-tagatose production, (b and e) d-fructose production, and (c and f) d-mannitol production. Error bars indicate s.d. (*n* = 3 biological replicates).

Ensuring production performance at high cell densities is vital for obtaining a comprehensive understanding of production capabilities.^57,58^ To address potential production constraints arising from glucose availability, AL4534 with pAL2606 (*P*_*gadB*_: gatZ-hxpA*) or pAL2607 (*P*_*gadB*_: kbaZ-hxpA*, Tables 1 and 2) were cultured under high cell density conditions for 24 h. Both these strains consumed 40 g L^–1^ glucose in 24 h (Figure S2). Based on this result, tagatose production at high cell density condition was tested with 40 g L^–1^ glucose feeding each day for 2 days. After the first 24 h, AL4534 with pAL2606 (*gatZ*) and pAL2607 (*kbaZ*) produced 3.2 g L^–1^ and 3.4 g L^–1^d-tagatose, respectively (Figure 6a). These strains also formed d-fructose and d-mannitol as side products (Figure 6b,c). A bolus of glucose was added to each culture, bringing the media glucose concentration back up to 40 g L^–1^. After an additional 24 h of culturing, AL4534 with pAL2606 (*gatZ*) and pAL2607 (*kbaZ*) produced 7.0 g L^–1^ and 8.7 g L^–1^d-tagatose, respectively (Figure 6a). d-fructose and d-mannitol production also increased (Figure 6a). This observation suggested the low C4 epimerase activity of GatZ and KbaZ causes the accumulated F6P to redirect the carbon flux toward d-fructose and d-mannitol. d-mannitol production is possible through the conversion of F6P to d-mannitol-1-phosphate (Mtl1P) via the enzyme mannitol-1-phosphate 5-dehydrogenase, encoded by the gene mtlD.

HxpA shows substrate promiscuity toward F6P and Mtl1P.^44,59^ To minimize the expression of *hxpA*, we reverted to the weaker *P*_LlacO1_ promoter plasmid system, pAL2574 (*P*_LlacO1_: gatZ-hxpA*) and pAL2575 (*P*_LlacO1_: kbaZ-hxpA*,Table 2). AL4534 with pAL2574 (*gatZ*) and pAL2575 (*kbaZ*) produced 4.5 g L^–1^ and 3.5 g L^–1^d-tagatose, respectively, after 24 h (Figure 6a). After a bolus of glucose and an additional 24 h, these strains produced 8.1 g L^–1^ and 7.6 g L^–1^d-tagatose, respectively. Additionally, 8.8 g L^–1^ and 7.8 g L^–1^d-fructose and 7.3 g L^–1^ and 7.1 g L^–1^d-mannitol were also produced (Figure 6b,c). Lowering the expression levels of *hxpA* resulted in similar d-tagatose titers, but d-fructose and d-mannitol were still produced.

### Carbon Starvation Strategy to Eliminate Side Product Formation

Strain AL4534 showed an inability to assimilate d-tagatose under the production conditions. Additionally, it has been shown that various *E. coli* strains cannot grow on d-tagatose,^60^ although *E. coli* is capable of redirecting phosphorylated d-tagatose toward glycolysis.^61^

Carbon starvation would be an effective approach to reduce side product formation for d-tagatose production. Under d-glucose-deprived conditions, the strain cannot assimilate the produced d-tagatose, ensuring its retention in the medium. Meanwhile, it can still consume other byproducts as alternative carbon sources, reducing sugar byproduct accumulation and enhancing product purity. This approach improves process selectivity and yield, making it a valuable strategy for optimizing microbial d-tagatose production. AL4534 with pAL2574 (*P*_LlacO1_, *gatZ*), pAL2575 (*P*_LlacO1_, *kbaZ*), pAL2606 (*P*_*gadB*_, *gatZ*), and pAL2607 (*P*_*gadB*_, *kbaZ*, Table 2) were employed for d-tagatose production under high-density conditions, supplemented with daily additions of 15 g L^–1^ glucose (Figure 6d–f). Within 24 h, the strains produced 1.5 g L^–1^, 1.2 g L^–1^, 1.4 g L^–1^, and 1.5 g L^–1^ of d-tagatose, respectively (Figure 6d). Notably, undetectable levels of d-fructose were formed, and AL4534 with pAL2574 (*P*_LlacO1_, *gatZ*), pAL2575 (*P*_LlacO1_, *kbaZ*) produced 1.2 g L^–1^ and 2.1 g L^–1^ of d-mannitol, respectively (Figure 6e,f). Upon additional glucose supplementation, d-tagatose titers increased to 4.6 g L^–1^, 4.5 g L^–1^, 4.7 g L^–1^, and 4.3 g L^–1^, respectively, after 48 h (Figure 6d). AL4534 with pAL2574 (*P*_LlacO1_, *gatZ*), pAL2575 (*P*_LlacO1_, *kbaZ*) utilized the produced d-mannitol. However, AL4534 with pAL2606 (*P*_*gadB*_, *gatZ*) and pAL2607 (*P*_*gadB*_, *kbaZ*) produced no d-mannitol, with 2.0 g L^–1^ and 0.7 g L^–1^ of d-fructose persisting in the media after 48 h (Figure 6e,f). Following another 15 g L^–1^ glucose addition and 78 h of cultivation, they produced 7.3 g L^–1^, 6.9 g L^–1^, 7.3 g L^–1^, and 7.3 g L^–1^ of d-tagatose, respectively (Figure 6d). While AL4534 with pAL2574 (*P*_LlacO1_, *gatZ*), pAL2575 (*P*_LlacO1_, *kbaZ*) produced undetectable side products, AL4534 with pAL2606 (*P*_*gadB*_, *gatZ*) and pAL2607 (*P*_*gadB*_, *kbaZ*) produced 5.2 g L^–1^ and 5.4 g L^–1^ of d-fructose, respectively (Figure 6e). The combination of a low glucose condition (15 g L^–1^)) and a weaker promoter (*P*_LlacO1_) enabled d-tagatose production without side products by the end of the production period. Although side products were initially formed, the strain efficiently consumed them, highlighting the effectiveness of our carbon starvation strategy in optimizing product formation.

Herein, our findings reveal that *E. coli*, with only the knockout of *pfkA* and *zwf* genes, possesses the inherent capacity to convert d-glucose into d-tagatose. We further demonstrate that GatZ and KbaZ are capable of converting F6P to d-tagatose-6-phosphate. Leveraging the substrate promiscuity of native phosphatases increased the level of d-tagatose production. By eliminating competing pathways and overexpression of native *E. coli* genes, we engineered a strain capable of d-tagatose production through a thermodynamically favorable biosynthetic pathway from easily accessible feedstocks. Our strain’s inability to utilize d-tagatose and their efficiency in consuming all d-glucose and side products present in the media reduce downstream purification requirements. Our results suggest that in addition to maintaining a low glucose concentration, carbon starvation during production prevents side product formation. Our final strain achieved conversion of 45 g L^–1^d-glucose to d-tagatose, reaching a titer of 7.3 g L^–1^, a productivity of 0.1 g L^–1^ h^–1^ without formation of any major side products.

## Data Availability

The data sets generated in this study are available from the corresponding author on reasonable request.

## Supporting Information Available

Table S1, which lists the oligonucleotides used in this study; Table S2, a plasmid construction guide; and Table S3, a guide for CRISPR-Cas9-mediated gene deletions and insertions; Table S4 presents the structural differences between the aldolase and epimerase enzyme families; Figure S1 shows the effect of gene deletion on d-mannose and d-psicose production, while Figure S2 illustrates glucose consumption of AL4534 under high-cell-density conditions; additionally, Rosetta XML files are provided (PDF)
