# Supplementary material for: Activating the d-Tagatose Production Capacity of Escherichia coli with Structural Insights into C4 Epimerase Specificity
Source: J Agric Food Chem. 2025 Feb 25;73(10):6124–34. doi: 10.1021/acs.jafc.4c12842 (PMC11907403; doi:10.1021/acs.jafc.4c12842)
Supplement: Supplementary file 1 — jf4c12842_si_001.pdf [file jf4c12842_si_001.pdf]

Supporting Information

Activating the D-Tagatose Production Capacity of *Escherichia coli* with Structural Insights into C4 Epimerase Specificity

Dileep Sai Kumar Palur[1], Jayce E. Taylor[1], Bryant Luu[2], Ian C. Anderson[3], Augustine Arredondo[1,6], Trevor Gannalo[2], Bryan A. Skorka[4], Pamela R. Denish[1], John Didzbalis[5], Justin B. Siegel[1,2,3,4,6,7], and Shota Atsumi[1,2*]

[1]Department of Chemistry, University of California, Davis, Davis, CA, 95616, USA

[2]Biochemistry, Molecular, Cellular, and Developmental Graduate Group, University of California, Davis, Davis, CA, 95616, USA

[3]Integrative Genetics and Genomics, University of California, Davis, Davis, CA, 95616, USA

[4]Biophysics Graduate Group, University of California, Davis, Davis, CA, 95616, USA

[5]Mars, Incorporated, 6885 Elm Street, McLean, VA 22101, USA

[6]Genome Center, University of California, Davis, Davis, CA 95616, USA.

[7]Department of Biochemistry and Molecular Medicine, University of California, Davis, Sacramento, CA 95616, USA.

*To whom correspondence may be addressed: E-mail: satsumi@ucdavis.edu

Table S1 Oligonucleotides used in this study

Name	Sequence 5′ to 3′	Plasmid(s) or fragment(s) produced
AZ0472	GGCTGCTAACAAAAGGAGATATACATATGAGCGTAAAAGTTATCGTCAC	pAL2492
AZ0482	CTGACCTGAATCAATTCAGCAGGAAGTGATTGTTATACTATTTGCACATTCGTTGGAT	Δ*pfkA* repair fragment (RF)
AZ0483	TCTGTTGCCGGAAGTCTTCTTGCACATCGAAGTGATCCAACGAATGTGCAAATAGTAT	Δ*pfkA* RF
AZ0484	AGACTTCCGGCAACAGATTTCATTTTGCATTCCAAAGTTCAGAGGTAGTCTGATTTCG	Δ*pfkA* RF
AZ0485	TGTCATCGGTTTCAGGGTAAAGGAATCTGCCTTTTTCCGAAATCAGACTACCTCTGAAC	Δ*pfkA* RF
AZ0486	ACCCTGAAACCGATGACAGAAGCAAAAATGCCTGATGCGCTTCGCTTATCAGGCCTACAT	Δ*pfkA* RF
AZ0487	CCTACAAAAGTTTGCAAATTCAATAAATTGCAGAATTCATGTAGGCCTGATAAGCGA	Δ*pfkA* RF
AZ0520	TCGGTCTGCCGTTTTAGAGCTAGAAATAGC	pAL1950
AZ0521	TGCACGGGAAACTAGTATTATACCTAGGAC	pAL1950
AZ0534	TGGGGATCGAGTTTTAGAGCTAGAAATAGC	pAL1958
AZ0535	CTTCTTCTGCACTAGTATTATACCTAGGAC	pAL1958
AZ0538	TAATCGCACGGGTGGATAAGCGTTTACAGTTTTCGCAAGCTCGTAAAAGCAGTACAGTGC	Δ*zwf* RF
AZ0539	CGGTACTTAAGCCAGGGTATACTTGTAATTTTCTTACGGTGCACTGTACTGCTTTTACGA	Δ*zwf* RF
AZ0540	ACCCTGGCTTAAGTACCGGGTTAGTTAACTTAAGGAGAATGACTATCTGCGCTTATCCT	Δ*zwf* RF
AZ0541	GCGCAAGATCATGTTACCGGTAAAATAACCATAAAGGATAAGCGCAGATAGTCATT	Δ*zwf* RF
AZ0542	CGGTAACATGATCTTGCGCAGATTGTAGAACAATTTTTACACTTTCAGGCCTCGTGCGGA	Δ*zwf* RF
AZ0543	CAGTCAGTGTAATAAAAAAAGCCTCGTGGGTGAATCCGCACGAGGCCTGAA	Δ*zwf* RF
AZ0667	GGTACCTTAGCAGCCGGATCTCAACCGAGAAGGTCTTTT	pAL2491
AZ0688	GATCCGGCTGCTAAGGTACCTAATCTAGAGGCATC	pAL2490, pAL2491, pAL2492, pAL2493, pAL2494, pAL2495, pAL2496
AZ0763	CAACACCAACACCGGCCGTAAATGCAGCATGATCGAACACATCATGCAGTC	Δ*pgm* RF
AZ0764	TCCGCAAACCTTCTCAATCAATTCCGCCGGGAATTGCATCGACTGCATGATGTGTTCGA	Δ*pgm* RF
AZ0765	GATTGAGAAGGTTTGCGGAACTATCTAAAACGTTGCAGACAAAGGACAAAGCAACACATT	Δ*pgm* RF
AZ0766	AAGGGCGATCTTGCGACCGCCCTTTTTTTATTAAATGTGTTGCTTTGTCCTTTGTCT	Δ*pgm* RF
AZ0767	CGCAAGATCGCCCTTTTTTACGTATGACAAACACAGAATTGCCTGATGCGCTACGCTTAT	Δ*pgm* RF
AZ0768	TCGCTTAAATTCAATATATTGCACCATCCTCGTAGGCCTGATAAGCGTAGCGCATCAG	Δ*pgm* RF
AZ0771	ACAATCGTGCGTTTTAGAGCTAGAAATAGC	pAL2038
AZ0772	GGATTGCCATACTAGTATTATACCTAGGAC	pAL2038
AZ1015	TTTTGTTAGCAGCCGGATCCTTACAGAACGTCGATCGCGTTCAG	pAL2586
AZ1024	GGATCCGGCTGCTAACAAAAGGAGATATACATATGTCAACCCCGCGTCAGATTCTTG	pAL2491
AZ1041	AATATCCTACACACTTTTTTAACAAAAACTGAGACTAGTACGACTTTTTGCGGCTCC	Δ*manA* RF
AZ1042	CCCACTATTAAAGCAAGAATCCTACGGGAAGTAACCTGGAGCCGCAAAAAGTCG	Δ*manA* RF
AZ1043	AGGATTCTTGCTTTAATAGTGGGATTAATTTCCACATTAAAACAGGGATTGATCGAGCT	Δ*manA* RF
AZ1044	GCCTTTAATAAGCTTAGCAAGAGATGTTAATTTTTTCAGTAAGCTCGATCAATCCCTGT	Δ*manA* RF
AZ1045	TCTCTTGCTAAGCTTATTAAAGGCTTATAACACCTTCAGGCGGCCAGTCCGCCTGATTTC	Δ*manA* RF
AZ1046	CTACCGCTACCAGCGATTTATTCATAATGATTATCCATAAAATGAAATCAGGCGGACTGG	Δ*manA* RF
BL007	TATTGCCCGTGTTTTAGAGCTAGAAATAGC	pAL2475
BL008	AAGGCCTTTTACTAGTATTATACCTAGGAC	pAL2475

BL009	CAGGGTTATCGACTAAGGATATTCAGCAAACCATCAAACTGG	Δ*gatZ* RF
BL010	AGGTTAGTCATATACCGTCCGTTTTTTCCTTGTTAAATGGC	Δ*gatZ* RF
BL011	GCCATTTAACAAGGAAAAAACGGACGGTATATGACTAACCT	Δ*gatZ* RF
BL012	CCAACGATACCGCCACGTCGTTATCATCA	Δ*gatZ* RF
BL013	AGGCGTGACGACTAGTATTATACCTAGGACTGAGC	pAL2481
BL014	TTGAAAAGCAGTTTTAGAGCTAGAAATAGCAAGTT	pAL2481
BL033	CGTTGCGGATCGTCACAGTAGATACGCCATACAATG	Δ*kbaZ* RF
BL034	CGTGTTCCTCTTTGTTTTGCGATAATGGTTTTCTGATCTG	Δ*kbaZ* RF
BL035	CAGATCAGAAAACCATTATCGCAAAACAAAGAGGAACACG	Δ*kbaZ* RF
BL036	GTCCAGAAACGTACGGCGATCCCTTCTG	Δ*kbaZ* RF
BL130	TTGATGGAACGCTGGTGGATTCCCTGC	pAL2574, pAL2575
BL131	GATCAAACAGAAAACCTTTGCACCGCATATGTATATCTCCTT	pAL2574, pAL2575
DS008	GGATCCGGCTGCTAACAAAAGGAGATATAC	pAL2480, pAL2497, pAL2586, pAL2587, pAL2588
DS016	TCGACGCTGAACTAGTATTATACCTAGGAC	pAL2190
DS017	GGCGGAGCTGGTTTTAGAGCTAGAAATAGC	pAL2190
DS018	CCACGCTGCAACTAGTATTATACCTAGGAC	pAL2189
DS019	AAGCAGAACTGTTTTAGAGCTAGAAATAGC	pAL2189
DS024	CGTTTGCACTGATGCAGTCGCTGCACATGGGC	Δ*kbaY* RF
DS025	CGGTGCCACTGGCTGATGGGGTATCCGGCGTATTGCCCATGTGCAGCGAC	Δ*kbaY* RF
DS026	GCCAGTGGCACCGTTAACCGCGTGGTGCAAG	Δ*kbaY* RF
DS027	TCCTCTTATGCCTGCCACGGATGAATGATTACGCCTTGCACCACGCGG	Δ*kbaY* RF
DS028	TGGCAGGCATAAGAGGATCGCATTTCATTAGTTTTTTATGATTTATCCCAATGTACTTC	Δ*kbaY* RF
DS029	AAGGCACTCCCCGATATTAATCGGGAAGTACATTGGGATAAATCATAAAAAACT	Δ*kbaY* RF
DS030	ATCGGGGAGTGCCTTAATGGAAAAGGAGATAACTAAACCTTAATAAATACATCACTAC	Δ*kbaY* RF
DS031	AAATATATTATTGTTGCGATATTGTAGTGATGTATTTATTAAGGTTTAGTTATCTCCTT	Δ*kbaY* RF
DS050	ATGTATATCTCCTTTTGTTAGCAGCCG	pAL2490, pAL2491, pAL2492, pAL2493, pAL2494, pAL2495, pAL2496
DS071	TCATGACGGCACTAGTATTATACCTAGGACTGAGCTAG	pAL2233
DS072	CATGAAAGTGGTTTTAGAGCTAGAAATAGCAAGTTAAAATAAG	pAL2233
DS075	CGCCATCATTGGCGGCACCAGTTTCTTCGGCGGCAAGGGGCGCATTTTCTCT	Δ*alsE* RF
DS076	GTTGTTGATGGTGCCGATGATCAACCCGCCAATCACCACAGAGAAAATGCGCCCCT	Δ*alsE* RF
DS077	TCGGCACCATCAACAACGGTCTGAATATTTTGCAGGTACAAACCTATTACCAACTGGTGG	Δ*alsE* RF
DS078	CAAGGGCGACAGCCGCGATAATTAATCCGCCCATCACCACCAGTTGGTAATAGGTTTG	Δ*alsE* RF
DS079	GGCTGTCGCCCTTGACCGTCTTATCAGTAAGTAAGGAATTGAACCGTCGTAGCGGGCGTG	Δ*alsE* RF
DS080	TGCTGTCCGCAGACAAAAGCGGATATGCGTTGCCCCCATATCCACGCCCGCTACG	Δ*alsE* RF
DS081	TGTCTGCGGACAGCAGAAGGTGAAACGCTACACTGCGAAAAAAAGCGGACCGCAGAAGTC	Δ*alsE* RF
DS082	AGGCCGGGAGCAATGACTTCTGCGGTCCGCTTTTTTTCGCAGTG	Δ*alsE* RF
DS137	CATTAAAGAGGAGAAAAGATATACCATGTCTAAGATTTTTGATTTCG	pAL2586
DS184	GGTATATCTTTTCTCCTCTTTAATGAATTC	pAL2480, pAL2497, pAL2586, pAL2587, pAL2588

DS316	TAACAAAAGGAGATATACATATGTACGAGCGTTATGCAGG	pAL2496
DS317	GGTACCTTAGCAGCCGGATCTCACAGCAAGCGAACATCCAC	pAL2496
DS318	TAACAAAAGGAGATATACATGTGCGGTGCAAAGGTTTTCTG	pAL2490
DS319	GGTACCTTAGCAGCCGGATCtcaCTGAATAATAACATCGC	pAL2490
DS320	GGTACCTTAGCAGCCGGATCTCAGCTGTTAAAAGGGGATG	pAL2492
DS321	TAACAAAAGGAGATATACATATGTACCAGGTTGTTGCGTC	pAL2494
DS322	GGTACCTTAGCAGCCGGATCTTACGATAAATAGAGTTTAC	pAL2494
DS324	GGTACCTTAGCAGCCGGATCTTAATTCAGCACATACTTCTC	pAL2493
DS325	TAACAAAAGGAGATATACATATGCTCTATATCTTTGATTTAG	pAL2495
DS326	AGGTACCTTAGCAGCCGGATCTTAGCATAACACCTTCGCG	pAL2495
DS496	GGCTGCTAACAAAAGGAGATATACATATGGCTATTAAACTCATTGCTATCG	pAL2493
DS538	AAGAGGAGAAAAGATATACCATGAAAACGTTAATTGCCCGGCATAAAG	pAL2480, pAL2606
DS539	TTTTGTTAGCAGCCGGATCCTTATTCCGCACAGCCGTAGCGATAGG	pAL2480
DS570	AAGAGGAGAAAAGATATACCGTGAAACATCTGACAGAAATGGTGA	pAL2497, pAL2607
DS571	CTCCTTTTGTTAGCAGCCGGATCCTTATTGGCCTTCACAGGCTGTGTGG	pAL2497
DS575	CCAGAGTCATACTAGTATTATACCTAGGACTGAGCTAG	pAL2568
DS576	AAATCGAACTGTTTTAGAGCTAGAAATAGCAAGTTAAAATAAG	pAL2568
DS577	AGGCGACAAGAGAGTGAATCCCCAGGAGCTTACATAAGTAAGTGACTGGGGTGAGCGAAC	Δ*fbaA* RF
DS578	CTCGTCATACTTCAAGTTGCATGTGCTGCGTCTGCGTTCGCTCACCCCAGT	Δ*fbaA* RF
DS579	TGCAACTTGAAGTATGACGAGTATAAGGCCCGACGATACAGGACAAGAGACGATATTCC	Δ*fbaA* RF
DS580	CCCGCAGAGCGGGCCTTGAGATAAGCAGAAAGGAATATCGTCTCTTGTCCTGT	Δ*fbaA* RF
DS581	CCCGCTCTGCGGGTCTTTTTTTCGCCAAAAGCAAAATTGCCTGATGCGCTACGCTTATCA	Δ*fbaA* RF
DS582	AACCTGCAAATTCAATATATTGCAGGATACGTGTAGGCCTGATAAGCGTAGCGCATC	Δ*fbaA* RF
DS617	TTAAAGAGGAGAAAAGATATACCATGTACGTGGTATCGACAAAG	pAL2587
DS618	TTTTGTTAGCAGCCGGATCCTTATGCCCTGCCCTCGCAGCCAC	pAL2587
DS619	AAGAGGAGAAAAGATATACCATGAGCATTATCTCCACTAAATATCTG	pAL2588
DS620	TTTTGTTAGCAGCCGGATCCTTATGCTGAAATTCGATTCGCTGAAC	pAL2588
JET011	GCCCTTTCGTCTTCACCTCGAGGTAATAATTTTATAAATGCG	pAL2606, pAL2607
JET018	CTCGAGGTGAAGACGAAAGGGCCTCGTGATACG	pAL2606, pAL2607
JET134	GGTATATCTTTTCTCCTCTTTAATGAATTCACTTGCTTCTTATCCATTTTAAACTCC	pAL2606, pAL2607
JET167	ATAGCCAGCAGTTTTAGAGCTAGAAATAGC	pAL2178
JET168	CGAGGGCCGAACTAGTATTATACCTAGGAC	pAL2178
JET250	CGTTTTTTTGCCTTTCACTCCTCGAATAATTTTCATATTGTCG	Δ*gatY* RF
JET251	TTGATTTTGCATATCCCCTTTCATATCCTGTCGTTTGTTTTCGATTTC	Δ*gatY* RF
JET252	AACGACAGGATATGAAAGGGGATATGCAAAATCAACGTTGCAACGGAGCTGAAAAATGCC	Δ*gatY* RF
JET253	TGCCGGGCAATTAACGTTTTCATGTTTTTTCCTTGTTAAATGGCAGGTGC	Δ*gatY* RF

Table S2 Plasmid construction guide

	Vector PCR			Insert(s) PCR			
Plasmid	Primer (F)	Primer (R)	Template	Primer (F)	Primer (R)	Template	Insert description
pAL2480	DS008	DS184	pAL2521	DS538	DS539	MG1655	*gatZ*
pAL2490				*DS318*	DS319	MG1655	*hxpA*
pAL2491				*AZ1024*	*AZ0667*	MG1655	*hxpB*
pAL2492				*AZ0472*	*DS320*	MG1655	*ybiV*
pAL2493				*DS496*	*DS324*	MG1655	*yidA*
pAL2494				*DS321*	*DS322*	MG1655	*yigL*
pAL2495				*DS325*	*DS326*	MG1655	*yihX*
pAL2496	DS668	DS050	pAL2480	*DS316*	*DS317*	MG1655	*yqaB*
pAL2497				DS570	DS571	MG1655	*kbaZ*
pAL2586				DS137	AZ1015	MG1655	*fbaA*
pAL2587				DS617	DS618	MG1655	*gatY*
pAL2588	DS008	DS184	pAL2490	DS619	DS620	MG1655	*kbaY*
pAL2574*	BL130	BL131	pAL2490				
pAL2575*	BL130	BL131	pAL2497				
pAL2606	DS538	JET018	pAL2474				
pAL2607	DS570	JET018	pAL2475	JET011	JET134	MG1655	P_{gadB}
pAL1950*	AZ0520	AZ0521					
pAL1958*	AZ0534	AZ0535					
pAL2475*	BL007	BL008					
pAL2481*	BL014	BL013					
pAL2568*	DS576	DS575					
pAL2190*	DS017	DS016					
pAL2189*	DS019	DS018					
pAL2233*	DS072	DS071					
pAL2178*	JET167	JET168					
pAL2038*	AZ0771	AZ0772	pTargetF				

*Q5-site directed mutagenesis (NEB)

Table S3 Guide for CRISPR-Cas9-mediate gene deletions and insertions

Modification	pTargetF		Linear repair fragment PCR primers
	Plasmid	20 bp sgRNA targeting sequence 5' to 3'	
Δ*pfkA*	pAL1950	TTCCCGTGCATCGGTCTGCC	AZ0482(F), AZ0483(R), AZ0484(F), AZ0485(R), AZ0486(F), AZ0487(R)
Δ*alsE*	pAL2233	GCCGTCATGACATGAAAGTG	DS075(F), DS076(R), DS078(F), DS079(R), DS080(F), DS081(R), DS082(F), DS083(R)
Δ*zwf*	pAL1958	GCAGAAGAAGTGGGGATCGA	AZ0538(F), AZ0539(R), AZ0540(F), AZ0541(R), AZ0542(F), AZ0543(R)
Δ*gatZ*	pAL2475	AAAAGGCCTTTATTGCCCGT	BL009(F), BL010(R), BL011(F), BL012(R)
Δ*manA*	pAL2178	TCGGCCCTCGATAGCCAGCA	AZ1041(F), AZ1042(R), AZ1043(F), AZ1044(R), AZ1045(F), AZ1046(R)
Δ*gatY*	pAL2190	TCAGCGTCGAGGCGGAGCTG	JET250(F), JET251(R), JET252(F), JET253(R)
Δ*kbaZ*	pAL2481	CGTCACGCCTTTGAAAAGCA	BL033(F), BL034(R), BL035(F), BL036(R)
Δ*kbaY*	pAL2189	TGCAGCGTGGAAGCAGAACT	DS024(F), DS025(R), DS026(F), DS027(R), DS028(F), DS029(R), DS030(F), DS031(R)
Δ*fbaA*	pAL2568	ATGACTCTGGAAATCGAACT	DS577(F), DS578(R), DS579(F), DS580(R), DS581(F), DS582(R)
Δ*pgm*	pAL2038	ATGGCAATCCACAATCGTGC	AZ0763(F), AZ0764(R), AZ0765(F), AZ0766(R), AZ0767(F), AZ0768(R)

Table S4. Structural difference between aldolase and epimerase enzyme family

GatZ			
**Residue**	**Position**	**Conservation**	**Function**
Glu	171	86.9	Zinc coordination
His	88	86.9	Zinc coordination
His	257	85.3	Zinc Coordination
Lys	279	64.4	Lost C3 Hydrogen Bonding
Glu	44	65	Lost C3 Hydrogen Bonding
Asp	87	87.1	Catalytic Acid Base
Cys	20	70.6	Catalytic Acid/Base Back up residue

FBA (3GAY)			
**Residue**	**Position**	**Conservation**	**Function**
His	178	78.08	Zinc coordination
His	84	79.63	Zinc coordination
His	210	81.01	Zinc Coordination
Asn	253	69.89	C3 Hydrogen Bonding
Gln	48	34.7	C3 Hydrogen Bonding
Asp	83	79.74	Catalytic Acid Base
Asn	24	66.57	Catalytic Acid/Base Back up residue

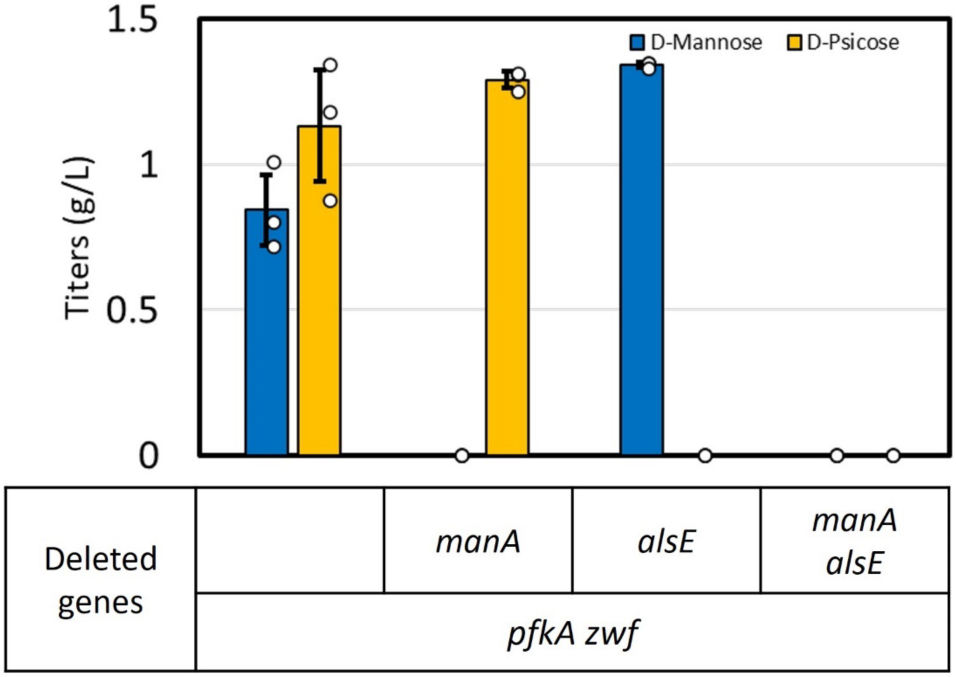

Figure S1. Effect of gene deletion on D-mannose and D-psicose production
Cells were grown in M9P media with 10 g L^{-1} glucose to OD_{600} ~0.4 at 37 °C, then grown at 30 ˚C for 24h. Each strain was transformed with pAL2606 (P_{gadB}: *gatZ-hxpA*). Error bars indicate s.d. (n = 3 biological replicates).

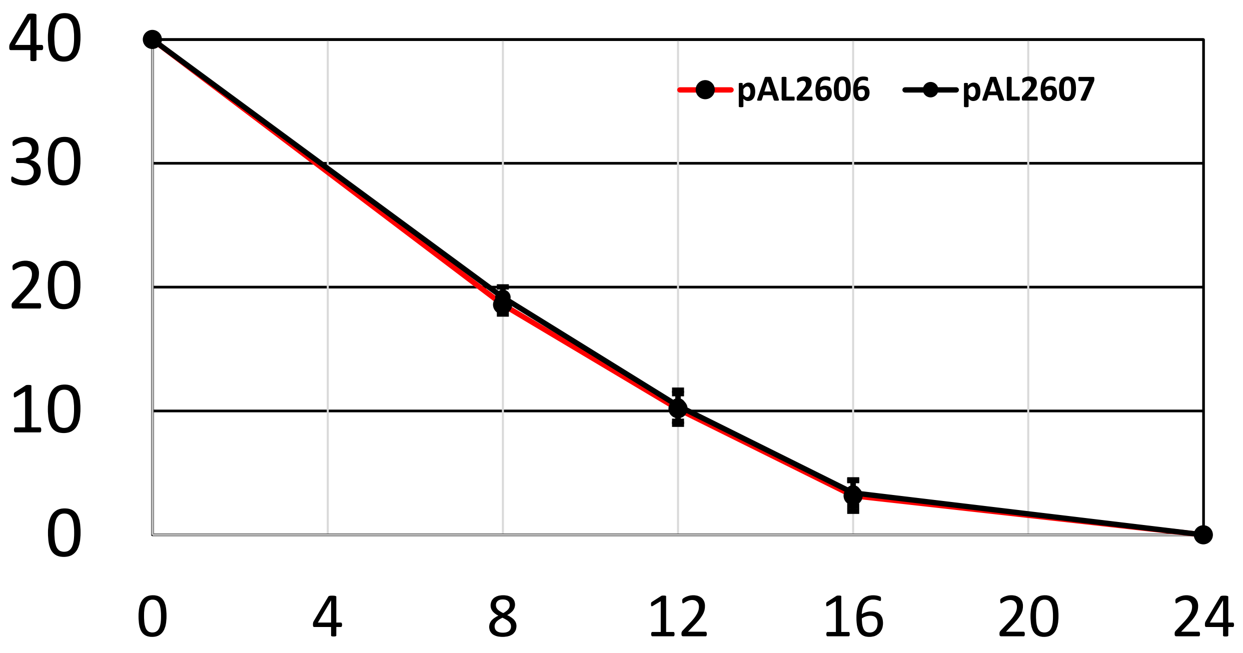

Figure S2. Glucose consumption of AL4534 at high cell density condition
Glucose consumption of AL4534 (**Table S1**) with pAL2606 or pAL2607 (**Table S2**). Cultures were grown in M9P media with 40 g L^{-1} glucose at 37 ˚C until an OD_{600} of ~0.4. Cultures were then spun down and resuspended in M9P media with 40 g L^{-1} glucose to an OD_{600} of ~10 and grown at 30 ˚C for 24 h. Error bars indicate s.d. (n = 3 biological replicates).

Rosetta XML:

All Rosetta simulations were run with Rosetta version 2018.24.post.dev+17.master.450949e

A detailed explanation with available sample data can be found on https://github.com/siegel-lab-ucd/D-tagatose-production

Commands:

~/Rosetta/main/source/bin/rosetta_scripts.default.linuxgccrelease -database ~/Rosetta/main/database, @flags -parser:protocol docking.xml -s GatZ_F6P.pdb -enzdes::cstfile 4Epimv7.cst -suffix -nstruct 1 -overwrite -suffix _$SLURM_ARRAY_TASK_ID -out:path:all test/

Example of the Rosetta script for docking(docking.xml)

```
<ROSETTASCRIPTS>
 <SCOREFXNS>
  <ScoreFunction name="myscore" weights="beta_genpot_cart.wts">
   <Reweight scoretype="coordinate_constraint" weight="1.0"/>
   <Reweight scoretype="atom_pair_constraint" weight="1.0"/>
   <Reweight scoretype="angle_constraint" weight="1.0"/>
   <Reweight scoretype="dihedral_constraint" weight="1.0"/>
   <Reweight scoretype="res_type_constraint" weight="1.0"/>
  </ScoreFunction>
  <ScoreFunction name="cstscore" weights="beta_genpot_cst.wts"/>

 </SCOREFXNS>
 <SCORINGGRIDS ligand_chain="X" width="20.0">
  <ClassicGrid grid_name="vdw" weight="1.0"/>
 </SCORINGGRIDS>
 <TASKOPERATIONS>
  <DetectProteinLigandInterface name="interface_detect" design="0" cut1="6.0"
cut2="8.0" cut3="10.0" cut4="12.0"/>
  <LimitAromaChi2 name="limchi2"/>
  <SetCatalyticResPackBehavior name="catres" fix_catalytic_aa="0"/>
  PreventResiduesFromRepacking name=nopacking residues=NOPACK />
 </TASKOPERATIONS>
 <FILTERS>
  <EnzScore name="allcst" score_type="cstE" scorefxn="cstscore" whole_pose="1"
energy_cutoff="2000"/>

 </FILTERS>
```

```
 <MOVERS>
  <AddOrRemoveMatchCsts name="cstadd" cst_instruction="add_new"/>

  <GALigandDock name="GAdock" runmode="refine" scorefxn="myscore" padding="6.0"
sidechains="aniso" final_exact_minimize="bbsc2" rotprob="0.9" rotEcut="100">
    Stage repeats="5" npool="50"/>
    <Stage repeats="5" npool="50"/>
  </GALigandDock>

 </MOVERS>
 <PROTOCOLS>
  CSTON  <Add mover_name="cstadd"/>
  Add mover="start_from"/>
  <Add mover="GAdock"/>
  Add mover="interative_dp"/>
  Add mover="repack_wbb_wppi"/>
  <Add filter="allcst"/>
 </PROTOCOLS>
</ROSETTASCRIPTS>
```

FLAGS:

```
-beta_cart
-run:preserve_header
-run:version
-nblist_autoupdate
-linmem_ig 10
-jd2::enzdes_out
-chemical:exclude_patches LowerDNA  UpperDNA Cterm_amidation VirtualBB ShoveBB
VirtualDNAPhosphate VirtualNTerm CTermConnect sc_orbitals pro_hydroxylated_case1
pro_hydroxylated_case2 ser_phosphorylated thr_phosphorylated  tyr_phosphorylated
tyr_sulfated lys_dimethylated lys_monomethylated  lys_trimethylated lys_acetylated
glu_carboxylated cys_acetylated tyr_diiodinated N_acetylated C_methylamidated
MethylatedProteinCterm

-enzdes::minimize_all_ligand_torsions 5.0
-enzdes::detect_design_interface


-packing::extrachi_cutoff 1
-packing::ex1
-packing::ex2
-packing::ex1aro:level 6
-packing::use_input_sc
```

```
-packing::flip_HNQ
-packing::no_optH false
-packing::optH_MCA false
-enzdes::favor_native_res 2
-enzdes::bb_min_allowed_dev 0.05

#-extra_res_fa 114.params
-extra_res_fa DF6.params
```

Catalytic Constraints

```
# Zn to His88
CST::BEGIN
 TEMPLATE::  ATOM_MAP: 1 atom_name: ZN V1 V2
 TEMPLATE::  ATOM_MAP: 1 residue3: ZN
 TEMPLATE::  ATOM_MAP: 2 atom_name: NE2 CE1 ND1
 TEMPLATE::  ATOM_MAP: 2 residue3: HIS
 CONSTRAINT::  distanceAB: 2.0     0.3 500.0 0
CST::END

# Zn to His257
CST::BEGIN
 TEMPLATE::  ATOM_MAP: 1 atom_name: ZN V1 V2
 TEMPLATE::  ATOM_MAP: 1 residue3: ZN
 TEMPLATE::  ATOM_MAP: 2 atom_name: NE2 CE1 ND1
 TEMPLATE::  ATOM_MAP: 2 residue3: HIS
 CONSTRAINT::  distanceAB: 2.0     0.3 500.0 0
CST::END


# Sugar to Asp 87 OD1
CST::BEGIN
 TEMPLATE::  ATOM_MAP: 1 atom_name: O3 C2 C3
 TEMPLATE::  ATOM_MAP: 1 residue3: DF6
 TEMPLATE::  ATOM_MAP: 2 atom_name: OD1 CG OD2
 TEMPLATE::  ATOM_MAP: 2 residue3: ASP
 CONSTRAINT::  distanceAB: 3.0 0.5 500.0 0
 CONSTRAINT::  angle_A: 113 5 50.0 180
#  CONSTRAINT::  angle_B: 120 20 50.0 180
#  CONSTRAINT::  torsion_A: 180 30 50.0 360
#  CONSTRAINT::  torsion_B: 180 30 50.0 360
CST::END
```

```
# Sugar O8 to ARG350  NH1
CST::BEGIN
  TEMPLATE::  ATOM_MAP: 1 atom_name: O8 P O5
  TEMPLATE::  ATOM_MAP: 1 residue3: DF6
  TEMPLATE::  ATOM_MAP: 2 atom_name: NH1 CZ NE
  TEMPLATE::  ATOM_MAP: 2 residue3: ARG
  CONSTRAINT::  distanceAB: 3.0 0.5 500.0 0
  CONSTRAINT::  angle_A: 120.9 10.0 50.0 180
  CONSTRAINT::  torsion_AB: 153.2 10 50.0 360
CST::END

# Sugar O7 to ASP260  NH1
CST::BEGIN
  TEMPLATE::  ATOM_MAP: 1 atom_name: O7 P O5
  TEMPLATE::  ATOM_MAP: 1 residue3: DF6
  TEMPLATE::  ATOM_MAP: 2 atom_name: OD2 CG OD1
  TEMPLATE::  ATOM_MAP: 2 residue3: ASP
  CONSTRAINT::  distanceAB: 3.0 0.5 500.0 0
  CONSTRAINT::  angle_A: 145 10.0 50.0 180
#  CONSTRAINT::  torsion_A: 180 30 50.0 360
CST::END

# Sugar O6 to GLY176  N
CST::BEGIN
  TEMPLATE::  ATOM_MAP: 1 atom_name: O6 P O5
  TEMPLATE::  ATOM_MAP: 1 residue3: DF6
  TEMPLATE::  ATOM_MAP: 2 atom_name: N
  TEMPLATE::  ATOM_MAP: 2 residue3: GLY
  CONSTRAINT::  distanceAB: 3.0 0.5 500.0 0
  CONSTRAINT::  angle_A: 145 10.0 50.0 180
#  CONSTRAINT::  torsion_A: 180 30 50.0 360
CST::END


# Zn to Sugar
CST::BEGIN
  TEMPLATE::  ATOM_MAP: 1 atom_name: ZN V1 V2
  TEMPLATE::  ATOM_MAP: 1 residue3: ZN
  TEMPLATE::  ATOM_MAP: 2 atom_name: O2 C3 C4
  TEMPLATE::  ATOM_MAP: 2 residue3: DF6
  CONSTRAINT::  distanceAB: 2.0     0.2 500.0 1
  CONSTRAINT::  angle_A: 123.2 5.0 100 180.0
CST::END
```

```
# Zn to Sugar
CST::BEGIN
  TEMPLATE::  ATOM_MAP: 1 atom_name: ZN V1 V2
  TEMPLATE::  ATOM_MAP: 1 residue3: ZN
  TEMPLATE::  ATOM_MAP: 2 atom_name: O C4 C3
  TEMPLATE::  ATOM_MAP: 2 residue3: DF6
  CONSTRAINT::  distanceAB: 2.0     0.2 500.0 1
  CONSTRAINT::  angle_A: 125.7 5.0 50 180.0
CST::END


# GLU255 to Sugar
CST::BEGIN
  TEMPLATE::  ATOM_MAP: 1 atom_name: O1 C5 C4
  TEMPLATE::  ATOM_MAP: 1 residue3: DF6
  TEMPLATE::  ATOM_MAP: 2 atom_name: OE1 CD OE2
  TEMPLATE::  ATOM_MAP: 2 residue3: GLU
  CONSTRAINT::  distanceAB: 3.0     0.5 500.0 0
#  CONSTRAINT::  angle_A: 125.0 10.0 50 180.0
CST::END

# ALA256to Sugar
CST::BEGIN
  TEMPLATE::  ATOM_MAP: 1 atom_name: O4 C1
  TEMPLATE::  ATOM_MAP: 1 residue3: DF6
  TEMPLATE::  ATOM_MAP: 2 atom_name: O C
  TEMPLATE::  ATOM_MAP: 2 residue3: ALA
  CONSTRAINT::  distanceAB: 3.0     0.5 500.0 0
#  CONSTRAINT::  angle_A: 125.0 5.0 50 180.0
CST::END
```

Ligand Params:

```
NAME DF6
IO_STRING DF6 Z
TYPE LIGAND
AA UNK
ATOM C    CSp  X    0.388
ATOM O    Oal  X   -0.572
ATOM P    PG3  X   -0.089
ATOM C1   CSp  X    0.061
ATOM O1   Ohx  X   -0.586
ATOM C2   CSp  X    0.147
ATOM O2   Ohx  X   -0.574
```

```
ATOM C3   CSp  X    0.085
ATOM O3   Ohx  X   -0.641
ATOM C4   CDp  X    0.559
ATOM O4   Ohx  X   -0.693
ATOM C5   CSp  X    0.093
ATOM O5   OG3  X   -0.612
ATOM O6   OG2  X   -0.639
ATOM O7   OG31 X   -0.611
ATOM O8   OG31 X   -0.611
ATOM H    HC   X   -0.001
ATOM H1   HC   X    0.016
ATOM H2   HC   X    0.064
ATOM H3   HC   X   -0.125
ATOM H4   HC   X   -0.125
ATOM H5   HC   X    0.035
ATOM H6   HC   X    0.035
ATOM H7   HO   X    0.394
ATOM H8   HO   X    0.400
ATOM H9   HO   X    0.427
ATOM H10  HO   X    0.459
ATOM H11  HO   X    0.359
ATOM H12  HO   X    0.359
BOND_TYPE C    C1   1
BOND_TYPE C    O5   1
BOND_TYPE P    O5   1
BOND_TYPE P    O7   1
BOND_TYPE P    O8   1
BOND_TYPE C1   C2   1
BOND_TYPE C1   O4   1
BOND_TYPE O1   C5   1
BOND_TYPE C2   C3   1
BOND_TYPE C2   O3   1
BOND_TYPE O2   C3   1
BOND_TYPE C3   C4   1
BOND_TYPE C4   C5   1
BOND_TYPE C    H3   1
BOND_TYPE C    H4   1
BOND_TYPE C1   H    1
BOND_TYPE O1   H7   1
BOND_TYPE C2   H1   1
BOND_TYPE O2   H8   1
BOND_TYPE C3   H2   1
BOND_TYPE O3   H9   1
BOND_TYPE O4   H10  1
```

```
BOND_TYPE C5   H5   1
BOND_TYPE C5   H6   1
BOND_TYPE O7   H11  1
BOND_TYPE O8   H12  1
BOND_TYPE O    C4   2
BOND_TYPE P    O6   2
NBR_ATOM C1
NBR_RADIUS 13.89632
ICOOR_INTERNAL  C1     0.000000    0.000000    0.000000 C1   C    O5
ICOOR_INTERNAL  C      0.000000  180.000000    1.529782 C1   C    O5
ICOOR_INTERNAL  O5     0.000000   70.506855    1.428471 C    C1   O5
ICOOR_INTERNAL  P    179.977649   56.996775    1.608978 O5   C    C1
ICOOR_INTERNAL  O7    64.951513   70.462066    1.610379 P    O5   C
ICOOR_INTERNAL  O8  -175.017637   70.503337    1.608879 P    O5   C
ICOOR_INTERNAL  C2  -174.959156   70.489107    1.530701 C1   C    O5
ICOOR_INTERNAL  O4    65.019859   70.518890    1.429423 C1   C    O5
ICOOR_INTERNAL  C3   179.984324   70.523469    1.529833 C2   C1   C
ICOOR_INTERNAL  O3    59.968276   70.536444    1.429423 C2   C1   C
ICOOR_INTERNAL  O2   -55.036499   70.534713    1.428731 C3   C2   C1
ICOOR_INTERNAL  C4  -175.046739   70.569305    1.507150 C3   C2   C1
ICOOR_INTERNAL  C5   -75.045311   59.942868    1.507154 C4   C3   C2
ICOOR_INTERNAL  O    105.008775   60.059342    1.208636 C4   C3   C2
ICOOR_INTERNAL  O6   -55.046363   70.516186    1.480968 P    O5   C
ICOOR_INTERNAL  O1   179.976719   70.503953    1.428673 C5   C4   C3
ICOOR_INTERNAL  H    -54.992559   70.466664    1.090489 C1   C    O5
ICOOR_INTERNAL  H1   -59.994630   70.582939    1.090765 C2   C1   C
ICOOR_INTERNAL  H2    64.988761   70.476347    1.089496 C3   C2   C1
ICOOR_INTERNAL  H3   119.987811   70.555176    1.090421 C    C1   O5
ICOOR_INTERNAL  H4  -120.082270   70.557147    1.091109 C    C1   O5
ICOOR_INTERNAL  H5    60.057222   70.608462    1.090164 C5   C4   C3
ICOOR_INTERNAL  H6   -59.966215   70.585787    1.088962 C5   C4   C3
ICOOR_INTERNAL  H7   179.977085   66.023892    0.967153 O1   C5   C4
ICOOR_INTERNAL  H8   -59.999647   65.999449    0.967219 O2   C3   C2
ICOOR_INTERNAL  H9    60.095854   66.008536    0.967066 O3   C2   C1
ICOOR_INTERNAL  H10   60.005448   66.053825    0.966178 O4   C1   C
ICOOR_INTERNAL  H11   59.934342   66.052735    0.967762 O7   P    O5
ICOOR_INTERNAL  H12  179.947373   65.933467    0.967585 O8   P    O5
CHI  1  C3  C4  C5  O1
CHI  2  C1   C  O5   P
CHI  3   C  C1  C2  C3
CHI  4   C  C1  O4 H10
PROTON_CHI 4 SAMPLES 3 60 -60 180 EXTRA 0
CHI  5   C  O5   P  O7
CHI  6  C2  C1   C  O5
```

CHI 7 O5 P O7 H11
PROTON_CHI 7 SAMPLES 3 60 -60 180 EXTRA 0
CHI 8 O5 P O8 H12
PROTON_CHI 8 SAMPLES 3 60 -60 180 EXTRA 0
CHI 9 C1 C2 C3 O2
CHI 10 C1 C2 O3 H9
PROTON_CHI 10 SAMPLES 3 60 -60 180 EXTRA 0
CHI 11 C2 C3 C4 C5
CHI 12 C2 C3 O2 H8
PROTON_CHI 12 SAMPLES 3 60 -60 180 EXTRA 0
CHI 13 C4 C5 O1 H7
PROTON_CHI 13 SAMPLES 3 60 -60 180 EXTRA 0
